# Supplementary material for: Assessment of Fatty Acid and Oxylipin Profile of Resprouting Olive Trees Positive to Xylella fastidiosa subsp. pauca in Salento (Apulia, Italy)
Source: Plants (Basel). 2024 Aug 7;13(16):2186. doi: 10.3390/plants13162186 (PMC11358993; doi:10.3390/plants13162186)

Supplementary Table S1. Summary of the NDVI index values calculated per each site and year considered for this analysis. I: XFP-positive symptomatic olive trees ; H: XFP-negative olive trees; R: XFP-positive resprouting olive trees.

Year	Site	Minimum	Maximum	Range	Mean
2018	H	0.075	0.202	0.12	0.16 ± 0.04
	I	0.016	0.127	0.11	0.09 ± 0.04
	R	0.011	0.146	0.13	0.1 ± 0.04
2019	H	0.09	0.166	0.07	0.13 ± 0.02
	I	0.001	0.125	0.12	0.07 ± 0.05
	R	0.028	0.192	0.16	0.13 ± 0.05
2020	H	0.098	0.2	0.1	0.16 ± 0.03
	I	0.009	0.139	0.13	0.09 ± 0.04
	R	0.085	0.213	0.12	0.18 ± 0.04
2021	H	0.106	0.185	0.07	0.15 ± 0.02
	I	0.07	0.149	0.07	0.11 ± 0.02
	R	0.054	0.195	0.14	0.15 ± 0.05
2022	H	0.073	0.169	0.09	0.11 ± 0.03
	I	0.044	0.101	0.05	0.07 ± 0.02
	R	0.059	0.208	0.14	0.13 ± 0.06

Supplementary Table S2. The normalized area of the FFAs in the samples analysed in this study. I, H and R indicate respectively: XFP-positive symptomatic olive trees (I); XFP-negative olive trees (H); XFP-positive resprouting olive trees (R).

Supplementary Table S3 _FFAs_normalized					
**Sample**	**variable**	**value**	**sd**	**Species**	**Condition**
cellina_infetti_200	16:0	43380,67	20875,76	Cellina	I
cellina_infetti_200	16:1	6721,474	2247,308	Cellina	I

cellina_infetti_200	18:0	56332,2	31060,98	Cellina	I
cellina_infetti_200	18:1	5382,133	1553,288	Cellina	I
cellina_infetti_200	18:2	34431,51	20397,57	Cellina	I
cellina_infetti_200	18:3	5641,372	3064,742	Cellina	I
cellina_infetti_200	20:4	3276,987	1039,885	Cellina	I
cellina_infetti_200	22:0	898413,6	281487,8	Cellina	I
cellina_infetti_200	24:0	27159,71	15433,42	Cellina	I
cellina_infetti_201	16:0	41038,27	9729,293	Cellina	I
cellina_infetti_201	16:1	6942,529	1446,371	Cellina	I
cellina_infetti_201	18:0	75207,89	54876,54	Cellina	I
cellina_infetti_201	18:1	4615,94	2006,552	Cellina	I
cellina_infetti_201	18:2	35370,4	13589,87	Cellina	I
cellina_infetti_201	18:3	5334,752	2443,936	Cellina	I
cellina_infetti_201	20:4	3059,057	1826,81	Cellina	I
cellina_infetti_201	22:0	857361,5	217345,2	Cellina	I
cellina_infetti_201	24:0	17063,93	11194,52	Cellina	I
cellina_infetti_208	16:0	5257,201	894,9044	Cellina	I
cellina_infetti_208	16:1	2145,119	215,654	Cellina	I
cellina_infetti_208	18:0	11198	3842,633	Cellina	I
cellina_infetti_208	18:1	1493,775	678,8891	Cellina	I
cellina_infetti_208	18:2	6337,506	1160,466	Cellina	I
cellina_infetti_208	18:3	7805,103	5024,294	Cellina	I
cellina_infetti_208	20:4	1269,634	385,0897	Cellina	I
cellina_infetti_208	22:0	272817,5	22602,96	Cellina	I
cellina_infetti_208	24:0	7816,778	1785,918	Cellina	I
cellina_infetti_209	16:0	13142,95	8783,166	Cellina	I
cellina_infetti_209	16:1	3888,451	1874,396	Cellina	I
cellina_infetti_209	18:0	19418,99	7497,522	Cellina	I
cellina_infetti_209	18:1	2061,523	897,5603	Cellina	I
cellina_infetti_209	18:2	10366,33	9071,462	Cellina	I
cellina_infetti_209	18:3	5239,055	2676,809	Cellina	I
cellina_infetti_209	20:4	2142,954	1120,444	Cellina	I
cellina_infetti_209	22:0	612318,9	218801,5	Cellina	I
cellina_infetti_209	24:0	10829,91	5514,614	Cellina	I
cellina_infetti_214	16:0	34818,59	1809,716	Cellina	I
cellina_infetti_214	16:1	6900,331	679,0109	Cellina	I
cellina_infetti_214	18:0	64994,44	34768,22	Cellina	I
cellina_infetti_214	18:1	4122,054	1962,645	Cellina	I
cellina_infetti_214	18:2	30754,84	23653,61	Cellina	I
cellina_infetti_214	18:3	4029,115	2699,809	Cellina	I
cellina_infetti_214	20:4	3421,959	3410,807	Cellina	I
cellina_infetti_214	22:0	753101,5	82200,26	Cellina	I
cellina_infetti_214	24:0	18186,21	9558,118	Cellina	I
cellina_infetti_217	16:0	41125,59	7337,587	Cellina	I
cellina_infetti_217	16:1	6213,528	1697,36	Cellina	I
cellina_infetti_217	18:0	122153,3	47856,96	Cellina	I

cellina_infetti_217	18:1	4099,956	1203,549	Cellina	I
cellina_infetti_217	18:2	32426,52	6942,879	Cellina	I
cellina_infetti_217	18:3	5957,429	1802,505	Cellina	I
cellina_infetti_217	20:4	1296,892	621,9121	Cellina	I
cellina_infetti_217	22:0	1063612	235219,2	Cellina	I
cellina_infetti_217	24:0	22931,98	11026,76	Cellina	I
cellina_Ri_184	16:0	23895,05	2644,443	Cellina	R
cellina_Ri_184	16:1	5604,078	776,3473	Cellina	R
cellina_Ri_184	18:0	41623,93	4095,119	Cellina	R
cellina_Ri_184	18:1	5263,679	891,6823	Cellina	R
cellina_Ri_184	18:2	30730,37	1676,999	Cellina	R
cellina_Ri_184	18:3	8506,327	2082,245	Cellina	R
cellina_Ri_184	20:4	3520,055	1372,612	Cellina	R
cellina_Ri_184	22:0	2127258	289682,5	Cellina	R
cellina_Ri_184	24:0	29276,48	5370,133	Cellina	R
cellina_Ri_185	16:0	27952,41	4211,055	Cellina	R
cellina_Ri_185	16:1	8256,114	1235,006	Cellina	R
cellina_Ri_185	18:0	31141,95	12575,5	Cellina	R
cellina_Ri_185	18:1	7161,007	1371,85	Cellina	R
cellina_Ri_185	18:2	39008,59	3840,533	Cellina	R
cellina_Ri_185	18:3	34783,82	5419,207	Cellina	R
cellina_Ri_185	20:4	3135,284	1227,801	Cellina	R
cellina_Ri_185	22:0	1774768	630204,1	Cellina	R
cellina_Ri_185	24:0	28124,97	9804,247	Cellina	R
cellina_Ri_186	16:0	26321,11	2001,189	Cellina	R
cellina_Ri_186	16:1	6842,742	399,3396	Cellina	R
cellina_Ri_186	18:0	53170,59	2094,914	Cellina	R
cellina_Ri_186	18:1	5898,916	1311,297	Cellina	R
cellina_Ri_186	18:2	34220,06	1849,587	Cellina	R
cellina_Ri_186	18:3	12059,76	2018,635	Cellina	R
cellina_Ri_186	20:4	3365,152	1078,395	Cellina	R
cellina_Ri_186	22:0	1656163	219785	Cellina	R
cellina_Ri_186	24:0	21693,28	6090,882	Cellina	R
cellina_Ri_187	16:0	13763,02	7340,796	Cellina	R
cellina_Ri_187	16:1	9191,601	643,8305	Cellina	R
cellina_Ri_187	18:0	17691,94	17949,3	Cellina	R
cellina_Ri_187	18:1	5561,809	1262,553	Cellina	R
cellina_Ri_187	18:2	53356,93	24828,07	Cellina	R
cellina_Ri_187	18:3	30966,27	4620,508	Cellina	R
cellina_Ri_187	20:4	4390,857	1762,281	Cellina	R
cellina_Ri_187	22:0	1884421	335791,5	Cellina	R
cellina_Ri_187	24:0	30542,59	9054,175	Cellina	R
cellina_Ri_196	16:0	81135,79	32719,11	Cellina	R
cellina_Ri_196	16:1	20788,03	9625,473	Cellina	R
cellina_Ri_196	18:0	83478,75	35107,4	Cellina	R
cellina_Ri_196	18:1	12924,54	6604,17	Cellina	R

cellina_Ri_196	18:2	308491,2	115265,6	Cellina	R
cellina_Ri_196	18:3	75523,49	39316,78	Cellina	R
cellina_Ri_196	20:4	12548,3	10139,95	Cellina	R
cellina_Ri_196	22:0	3104918	1677561	Cellina	R
cellina_Ri_196	24:0	52609,73	39031,21	Cellina	R
cellina_Ri_197	16:0	92068,49	92536,62	Cellina	R
cellina_Ri_197	16:1	17227,82	13724,16	Cellina	R
cellina_Ri_197	18:0	82383,19	89079,02	Cellina	R
cellina_Ri_197	18:1	7973,437	8831,085	Cellina	R
cellina_Ri_197	18:2	104448,9	121606,3	Cellina	R
cellina_Ri_197	18:3	25270,82	25928,76	Cellina	R
cellina_Ri_197	20:4	29353,98	34514,94	Cellina	R
cellina_Ri_197	22:0	2640609	2268805	Cellina	R
cellina_Ri_197	24:0	27063,68	28168,44	Cellina	R
cellina_sani_169	16:0	19870,17	1843,516	Cellina	H
cellina_sani_169	16:1	10635,73	1635,177	Cellina	H
cellina_sani_169	18:0	9947,659	2817,411	Cellina	H
cellina_sani_169	18:1	10103,73	1259,433	Cellina	H
cellina_sani_169	18:2	127613,9	62317,5	Cellina	H
cellina_sani_169	18:3	33238,83	10332,38	Cellina	H
cellina_sani_169	20:4	3541,465	1406,961	Cellina	H
cellina_sani_169	22:0	1315091	154530,3	Cellina	H
cellina_sani_169	24:0	20435,03	7536,233	Cellina	H
cellina_sani_170	16:0	21803,25	1265,652	Cellina	H
cellina_sani_170	16:1	10816,35	1409,28	Cellina	H
cellina_sani_170	18:0	12574,49	3017,771	Cellina	H
cellina_sani_170	18:1	7892,089	1516,463	Cellina	H
cellina_sani_170	18:2	55645,08	11444,62	Cellina	H
cellina_sani_170	18:3	58857,62	4017,404	Cellina	H
cellina_sani_170	20:4	4255,782	1364,52	Cellina	H
cellina_sani_170	22:0	2379049	235749,4	Cellina	H
cellina_sani_170	24:0	42131,82	2626,541	Cellina	H
cellina_sani_171	16:0	20097,05	2732,737	Cellina	H
cellina_sani_171	16:1	23237,69	2425,629	Cellina	H
cellina_sani_171	18:0	13038,14	4637,967	Cellina	H
cellina_sani_171	18:1	8508,991	1049,559	Cellina	H
cellina_sani_171	18:2	60030,32	6631,211	Cellina	H
cellina_sani_171	18:3	50776,69	14562,27	Cellina	H
cellina_sani_171	20:4	4072,547	825,993	Cellina	H
cellina_sani_171	22:0	1602091	415300	Cellina	H
cellina_sani_171	24:0	24762,25	6194,346	Cellina	H
cellina_sani_172	16:0	35766,83	9420,472	Cellina	H
cellina_sani_172	16:1	11279,6	1476,319	Cellina	H
cellina_sani_172	18:0	40888,13	19745,36	Cellina	H
cellina_sani_172	18:1	8143,602	2521,462	Cellina	H
cellina_sani_172	18:2	83143,06	19247,29	Cellina	H

cellina_sani_172	18:3	83532,98	9742,749	Cellina	H
cellina_sani_172	20:4	4734,668	1629,204	Cellina	H
cellina_sani_172	22:0	2848288	362102	Cellina	H
cellina_sani_172	24:0	47930,05	3995,244	Cellina	H
cellina_sani_176	16:0	19063,89	4053,426	Cellina	H
cellina_sani_176	16:1	4164,442	970,4894	Cellina	H
cellina_sani_176	18:0	25760,39	6197,312	Cellina	H
cellina_sani_176	18:1	2924,094	1676,282	Cellina	H
cellina_sani_176	18:2	20123,18	5406,796	Cellina	H
cellina_sani_176	18:3	3823,389	1146,377	Cellina	H
cellina_sani_176	20:4	3715,731	2298,656	Cellina	H
cellina_sani_176	22:0	935108,7	266505,5	Cellina	H
cellina_sani_176	24:0	29790,34	11277,46	Cellina	H
cellina_sani_179	16:0	34356,56	18120,41	Cellina	H
cellina_sani_179	16:1	8467,095	3296,352	Cellina	H
cellina_sani_179	18:0	43207,64	25312,25	Cellina	H
cellina_sani_179	18:1	4397,188	1381,324	Cellina	H
cellina_sani_179	18:2	34839,86	16921,7	Cellina	H
cellina_sani_179	18:3	4951,9	1989,72	Cellina	H
cellina_sani_179	20:4	5124,52	3132,4	Cellina	H
cellina_sani_179	22:0	1045319	438893,3	Cellina	H
cellina_sani_179	24:0	29563,85	19821,23	Cellina	H
ogliarola_infetti_202	16:0	100281,6	6581,085	Ogliarola	I
ogliarola_infetti_202	16:1	12557,56	1497,098	Ogliarola	I
ogliarola_infetti_202	18:0	36464,7	9975,928	Ogliarola	I
ogliarola_infetti_202	18:1	10563,74	1350,235	Ogliarola	I
ogliarola_infetti_202	18:2	163890,7	17154,33	Ogliarola	I
ogliarola_infetti_202	18:3	67800,78	13558,51	Ogliarola	I
ogliarola_infetti_202	20:4	4584,914	2290,758	Ogliarola	I
ogliarola_infetti_202	22:0	1864555	731356,9	Ogliarola	I
ogliarola_infetti_202	24:0	24056,1	7247,788	Ogliarola	I
ogliarola_infetti_203	16:0	114264,5	3803,471	Ogliarola	I
ogliarola_infetti_203	16:1	13046,21	1644,771	Ogliarola	I
ogliarola_infetti_203	18:0	46878,44	11532,82	Ogliarola	I
ogliarola_infetti_203	18:1	11843,66	1085,332	Ogliarola	I
ogliarola_infetti_203	18:2	238110,4	18451,82	Ogliarola	I
ogliarola_infetti_203	18:3	67435,4	8205,936	Ogliarola	I
ogliarola_infetti_203	20:4	3562,002	1407,515	Ogliarola	I
ogliarola_infetti_203	22:0	2168721	401674,4	Ogliarola	I
ogliarola_infetti_203	24:0	27811,02	5066,489	Ogliarola	I
ogliarola_infetti_206	16:0	71633,44	136860,9	Ogliarola	I
ogliarola_infetti_206	16:1	26727,26	52853	Ogliarola	I
ogliarola_infetti_206	18:0	23117,19	25073,5	Ogliarola	I
ogliarola_infetti_206	18:1	40767,78	69898,7	Ogliarola	I
ogliarola_infetti_206	18:2	136010,9	264127,6	Ogliarola	I
ogliarola_infetti_206	18:3	28839,97	46977,97	Ogliarola	I

ogliarola_infetti_206	20:4	38837,29	30878	Ogliarola	I
ogliarola_infetti_206	22:0	3750507	6661875	Ogliarola	I
ogliarola_infetti_206	24:0	30797,2	20202,26	Ogliarola	I
ogliarola_infetti_207	16:0	142568,5	159002,8	Ogliarola	I
ogliarola_infetti_207	16:1	29933,6	27382,26	Ogliarola	I
ogliarola_infetti_207	18:0	64920,73	64839,24	Ogliarola	I
ogliarola_infetti_207	18:1	24733,7	26232,18	Ogliarola	I
ogliarola_infetti_207	18:2	131261,2	163289,5	Ogliarola	I
ogliarola_infetti_207	18:3	65864,94	56846,3	Ogliarola	I
ogliarola_infetti_207	20:4	41909,24	64583,67	Ogliarola	I
ogliarola_infetti_207	22:0	5401315	4165079	Ogliarola	I
ogliarola_infetti_207	24:0	72712,67	69923,51	Ogliarola	I
ogliarola_infetti_213	16:0	359977,5	299782	Ogliarola	I
ogliarola_infetti_213	16:1	32639,59	29603,7	Ogliarola	I
ogliarola_infetti_213	18:0	106867,6	102727,6	Ogliarola	I
ogliarola_infetti_213	18:1	26013,01	18112,33	Ogliarola	I
ogliarola_infetti_213	18:2	155443,1	109133,2	Ogliarola	I
ogliarola_infetti_213	18:3	292265,9	228583,7	Ogliarola	I
ogliarola_infetti_213	20:4	31852,53	37812,15	Ogliarola	I
ogliarola_infetti_213	22:0	8353091	7125470	Ogliarola	I
ogliarola_infetti_213	24:0	98405,41	85098,68	Ogliarola	I
ogliarola_infetti_216	16:0	28677,79	4434,663	Ogliarola	I
ogliarola_infetti_216	16:1	5709,821	599,7869	Ogliarola	I
ogliarola_infetti_216	18:0	92493,93	22516,97	Ogliarola	I
ogliarola_infetti_216	18:1	4171,516	1122,245	Ogliarola	I
ogliarola_infetti_216	18:2	45910,3	32617,3	Ogliarola	I
ogliarola_infetti_216	18:3	10106,39	9424,793	Ogliarola	I
ogliarola_infetti_216	20:4	923,7266	285,4186	Ogliarola	I
ogliarola_infetti_216	22:0	921908,2	176440,8	Ogliarola	I
ogliarola_infetti_216	24:0	12017,52	6817,824	Ogliarola	I
ogliarola_Ri_190	16:0	69765,9	24046,25	Ogliarola	R
ogliarola_Ri_190	16:1	7722,714	2479,967	Ogliarola	R
ogliarola_Ri_190	18:0	61940	25530	Ogliarola	R
ogliarola_Ri_190	18:1	11800,06	4840,997	Ogliarola	R
ogliarola_Ri_190	18:2	90323,33	41581,88	Ogliarola	R
ogliarola_Ri_190	18:3	53775,18	16759,47	Ogliarola	R
ogliarola_Ri_190	20:4	5646,588	2512,413	Ogliarola	R
ogliarola_Ri_190	22:0	1969225	1394122	Ogliarola	R
ogliarola_Ri_190	24:0	21784,43	12843,44	Ogliarola	R
ogliarola_Ri_191	16:0	55673,16	13594,01	Ogliarola	R
ogliarola_Ri_191	16:1	6697,361	604,1213	Ogliarola	R
ogliarola_Ri_191	18:0	59770,67	12772,78	Ogliarola	R
ogliarola_Ri_191	18:1	8928,285	1775,774	Ogliarola	R
ogliarola_Ri_191	18:2	151701,7	36482,87	Ogliarola	R
ogliarola_Ri_191	18:3	55109,04	13024,34	Ogliarola	R
ogliarola_Ri_191	20:4	4722,683	1880,302	Ogliarola	R

ogliarola_Ri_191	22:0	3414281	916040,1	Ogliarola	R
ogliarola_Ri_191	24:0	33935,88	16963,39	Ogliarola	R
ogliarola_Ri_192	16:0	30590,65	3565,625	Ogliarola	R
ogliarola_Ri_192	16:1	8627,285	1265,923	Ogliarola	R
ogliarola_Ri_192	18:0	28803,18	4201,535	Ogliarola	R
ogliarola_Ri_192	18:1	8821,716	1096,358	Ogliarola	R
ogliarola_Ri_192	18:2	98979,9	28395,43	Ogliarola	R
ogliarola_Ri_192	18:3	30269,74	8201,515	Ogliarola	R
ogliarola_Ri_192	20:4	3796,908	1541,012	Ogliarola	R
ogliarola_Ri_192	22:0	2294602	305326,4	Ogliarola	R
ogliarola_Ri_192	24:0	28612,92	6013,461	Ogliarola	R
ogliarola_Ri_193	16:0	93589,32	9242,534	Ogliarola	R
ogliarola_Ri_193	16:1	8674,864	1228,419	Ogliarola	R
ogliarola_Ri_193	18:0	41616,09	15091,65	Ogliarola	R
ogliarola_Ri_193	18:1	7807,548	963,9104	Ogliarola	R
ogliarola_Ri_193	18:2	94629,03	19534,9	Ogliarola	R
ogliarola_Ri_193	18:3	35301,88	9820,601	Ogliarola	R
ogliarola_Ri_193	20:4	2389,249	1546,251	Ogliarola	R
ogliarola_Ri_193	22:0	1450142	148540	Ogliarola	R
ogliarola_Ri_193	24:0	17333,06	2171,774	Ogliarola	R
ogliarola_Ri_194	16:0	86504,49	48110,34	Ogliarola	R
ogliarola_Ri_194	16:1	14666,04	5548,996	Ogliarola	R
ogliarola_Ri_194	18:0	94828,38	57930,71	Ogliarola	R
ogliarola_Ri_194	18:1	20934,65	33847,97	Ogliarola	R
ogliarola_Ri_194	18:2	145023,6	55977,47	Ogliarola	R
ogliarola_Ri_194	18:3	41206,24	26990,64	Ogliarola	R
ogliarola_Ri_194	20:4	33293,66	27663,73	Ogliarola	R
ogliarola_Ri_194	22:0	2573678	876660,9	Ogliarola	R
ogliarola_Ri_194	24:0	45472,54	20002,56	Ogliarola	R
ogliarola_Ri_195	16:0	196441,5	249759,5	Ogliarola	R
ogliarola_Ri_195	16:1	25015,83	21319,7	Ogliarola	R
ogliarola_Ri_195	18:0	257843,7	270165,4	Ogliarola	R
ogliarola_Ri_195	18:1	23561,25	29166,54	Ogliarola	R
ogliarola_Ri_195	18:2	277532,3	273102,9	Ogliarola	R
ogliarola_Ri_195	18:3	88035,95	78520,05	Ogliarola	R
ogliarola_Ri_195	20:4	21795,36	17262,13	Ogliarola	R
ogliarola_Ri_195	22:0	3125424	2521989	Ogliarola	R
ogliarola_Ri_195	24:0	68419,31	63242,64	Ogliarola	R
ogliarola_sana_154	16:0	66649,17	20174,73	Ogliarola	H
ogliarola_sana_154	16:1	10108,87	4398,721	Ogliarola	H
ogliarola_sana_154	18:0	25150,55	5522,352	Ogliarola	H
ogliarola_sana_154	18:1	10648,62	7671,026	Ogliarola	H
ogliarola_sana_154	18:2	139556,3	23007,31	Ogliarola	H
ogliarola_sana_154	18:3	5664,621	1687,155	Ogliarola	H
ogliarola_sana_154	20:4	1190,605	732,2214	Ogliarola	H
ogliarola_sana_154	22:0	2713321	437434,9	Ogliarola	H

ogliarola_sana_154	24:0	16172,3	16508,91	Ogliarola	H
ogliarola_sana_155	16:0	96450,24	11196,34	Ogliarola	H
ogliarola_sana_155	16:1	5423,25	907,3423	Ogliarola	H
ogliarola_sana_155	18:0	32790,4	11730,9	Ogliarola	H
ogliarola_sana_155	18:1	6939,197	1122,527	Ogliarola	H
ogliarola_sana_155	18:2	153495,7	14200,63	Ogliarola	H
ogliarola_sana_155	18:3	21833,41	13149,53	Ogliarola	H
ogliarola_sana_155	20:4	1859,104	1189,33	Ogliarola	H
ogliarola_sana_155	22:0	3615772	721533,9	Ogliarola	H
ogliarola_sana_155	24:0	15867,99	9619,147	Ogliarola	H
ogliarola_sana_156	16:0	162839,1	38254,3	Ogliarola	H
ogliarola_sana_156	16:1	8619,343	2185,856	Ogliarola	H
ogliarola_sana_156	18:0	83745,95	37885,21	Ogliarola	H
ogliarola_sana_156	18:1	13463,89	7297,677	Ogliarola	H
ogliarola_sana_156	18:2	316114	82965,96	Ogliarola	H
ogliarola_sana_156	18:3	80361,13	22687,01	Ogliarola	H
ogliarola_sana_156	20:4	4146,459	1856,765	Ogliarola	H
ogliarola_sana_156	22:0	5428096	1646808	Ogliarola	H
ogliarola_sana_156	24:0	48993,44	19776,6	Ogliarola	H
ogliarola_sana_157	16:0	94928,49	89302,38	Ogliarola	H
ogliarola_sana_157	16:1	8544,296	4665,723	Ogliarola	H
ogliarola_sana_157	18:0	80126,02	51308,72	Ogliarola	H
ogliarola_sana_157	18:1	22023,84	8805,907	Ogliarola	H
ogliarola_sana_157	18:2	186167,3	119855	Ogliarola	H
ogliarola_sana_157	18:3	89425,42	48179,04	Ogliarola	H
ogliarola_sana_157	20:4	3126,677	1185,67	Ogliarola	H
ogliarola_sana_157	22:0	5824769	1781861	Ogliarola	H
ogliarola_sana_157	24:0	79364,63	42137,57	Ogliarola	H
ogliarola_sana_158	16:0	13853,48	3971,748	Ogliarola	H
ogliarola_sana_158	16:1	9764,151	1035,272	Ogliarola	H
ogliarola_sana_158	18:0	11504,45	4242,887	Ogliarola	H
ogliarola_sana_158	18:1	9571,368	2510,504	Ogliarola	H
ogliarola_sana_158	18:2	71326,24	23349,62	Ogliarola	H
ogliarola_sana_158	18:3	12817,66	2689,934	Ogliarola	H
ogliarola_sana_158	20:4	8944,039	6941,688	Ogliarola	H
ogliarola_sana_158	22:0	3100035	387024,4	Ogliarola	H
ogliarola_sana_158	24:0	52450,35	21330,02	Ogliarola	H
ogliarola_sana_159	16:0	43332,94	81113,86	Ogliarola	H
ogliarola_sana_159	16:1	16574,58	19140,91	Ogliarola	H
ogliarola_sana_159	18:0	26986,63	45876,77	Ogliarola	H
ogliarola_sana_159	18:1	12204,36	7941,584	Ogliarola	H
ogliarola_sana_159	18:2	106909,6	85457,08	Ogliarola	H
ogliarola_sana_159	18:3	19450,76	9538,446	Ogliarola	H
ogliarola_sana_159	20:4	7978,765	7861,134	Ogliarola	H
ogliarola_sana_159	22:0	4090099	4726235	Ogliarola	H
ogliarola_sana_159	24:0	64038,64	56852,01	Ogliarola	H

ogliarola_sana_160	16:0	23718,83	4279,634	Ogliarola	H
ogliarola_sana_160	16:1	8211,8	1377,773	Ogliarola	H
ogliarola_sana_160	18:0	17473,8	6509,152	Ogliarola	H
ogliarola_sana_160	18:1	4753,813	1651,668	Ogliarola	H
ogliarola_sana_160	18:2	83419,3	62659,4	Ogliarola	H
ogliarola_sana_160	18:3	12765,35	5691,6	Ogliarola	H
ogliarola_sana_160	20:4	4785,393	1223,801	Ogliarola	H
ogliarola_sana_160	22:0	1762807	263547,1	Ogliarola	H
ogliarola_sana_160	24:0	29022,06	13867,07	Ogliarola	H
ogliarola_sana_161	16:0	16258,57	8452,248	Ogliarola	H
ogliarola_sana_161	16:1	6487,844	6820,581	Ogliarola	H
ogliarola_sana_161	18:0	15102,55	21035,19	Ogliarola	H
ogliarola_sana_161	18:1	11008,88	25404,13	Ogliarola	H
ogliarola_sana_161	18:2	54602,02	30256,26	Ogliarola	H
ogliarola_sana_161	18:3	18145,14	11853,55	Ogliarola	H
ogliarola_sana_161	20:4	3165,614	1632,808	Ogliarola	H
ogliarola_sana_161	22:0	1572403	1275526	Ogliarola	H
ogliarola_sana_161	24:0	16680,18	15856,6	Ogliarola	H

Supplementary Table S3. The normalized area of the oxylipins and hormones in the samples analysed in this study. I, H and R indicate respectively: XFP-positive symptomatic olive trees (I); XFP-negative olive trees (H); XFP-positive resprouting olive trees (R).

**Supplementary_Table_S4_oxylipin and_Hormones_normalized**				
**Sample**	**Oxy**	**value**	**condition**	**species**
cellina_infetti_200	10-HODE	2084,068739	I	Cellina
cellina_infetti_201	10-HODE	1258,85965	I	Cellina
cellina_infetti_208	10-HODE	2197,699405	I	Cellina
cellina_infetti_209	10-HODE	244,1827386	I	Cellina
cellina_infetti_214	10-HODE	1702,130202	I	Cellina
cellina_infetti_217	10-HODE	1079,481205	I	Cellina
cellina_Ri_184	10-HODE	2016,005132	R	Cellina
cellina_Ri_185	10-HODE	1851,373627	R	Cellina
cellina_Ri_186	10-HODE	1381,993252	R	Cellina
cellina_Ri_187	10-HODE	2034,551089	R	Cellina
cellina_Ri_196	10-HODE	27388,49857	R	Cellina
cellina_Ri_197	10-HODE	2493,427335	R	Cellina
cellina_sani_169	10-HODE	1530,257804	H	Cellina
cellina_sani_170	10-HODE	1570,177515	H	Cellina
cellina_sani_171	10-HODE	3043,662581	H	Cellina
cellina_sani_172	10-HODE	4112,958647	H	Cellina
cellina_sani_176	10-HODE	2085,36621	H	Cellina
cellina_sani_179	10-HODE	4380,477287	H	Cellina
ogliarola_infetti_202	10-HODE	1107,689878	I	Ogliarola
ogliarola_infetti_203	10-HODE	700,9361104	I	Ogliarola
ogliarola_infetti_206	10-HODE	3367,228951	I	Ogliarola

ogliarola_infetti_207	10-HODE	7754,629239	I	Ogliarola
ogliarola_infetti_213	10-HODE	13848,27255	I	Ogliarola
ogliarola_infetti_216	10-HODE	2214,248651	I	Ogliarola
ogliarola_Ri_190	10-HODE	1028,404829	R	Ogliarola
ogliarola_Ri_191	10-HODE	958,0412828	R	Ogliarola
ogliarola_Ri_192	10-HODE	1316,890826	R	Ogliarola
ogliarola_Ri_193	10-HODE	464,8475203	R	Ogliarola
ogliarola_Ri_194	10-HODE	3593,548914	R	Ogliarola
ogliarola_Ri_195	10-HODE	3830,906782	R	Ogliarola
ogliarola_sana_154	10-HODE	22693,74119	H	Ogliarola
ogliarola_sana_155	10-HODE	3060,689154	H	Ogliarola
ogliarola_sana_156	10-HODE	4406,501713	H	Ogliarola
ogliarola_sana_157	10-HODE	4427,923285	H	Ogliarola
ogliarola_sana_158	10-HODE	5935,262239	H	Ogliarola
ogliarola_sana_159	10-HODE	4301,532423	H	Ogliarola
cellina_infetti_200	10-HpOME	17132,61031	I	Cellina
cellina_infetti_201	10-HpOME	13784,05879	I	Cellina
cellina_infetti_208	10-HpOME	7890,661762	I	Cellina
cellina_infetti_209	10-HpOME	13086,75577	I	Cellina
cellina_infetti_214	10-HpOME	11033,99846	I	Cellina
cellina_infetti_217	10-HpOME	15853,05819	I	Cellina
cellina_Ri_184	10-HpOME	36353,80811	R	Cellina
cellina_Ri_185	10-HpOME	32620,84299	R	Cellina
cellina_Ri_186	10-HpOME	24852,36494	R	Cellina
cellina_Ri_187	10-HpOME	28578,37982	R	Cellina
cellina_Ri_196	10-HpOME	73491,4316	R	Cellina
cellina_Ri_197	10-HpOME	60451,80627	R	Cellina
cellina_sani_169	10-HpOME	21172,72689	H	Cellina
cellina_sani_170	10-HpOME	36441,1971	H	Cellina
cellina_sani_171	10-HpOME	26051,99486	H	Cellina
cellina_sani_172	10-HpOME	55211,84446	H	Cellina
cellina_sani_176	10-HpOME	25658,25953	H	Cellina
cellina_sani_179	10-HpOME	22879,27947	H	Cellina
ogliarola_infetti_202	10-HpOME	29654,70912	I	Ogliarola
ogliarola_infetti_203	10-HpOME	28099,01256	I	Ogliarola
ogliarola_infetti_206	10-HpOME	38523,03812	I	Ogliarola
ogliarola_infetti_207	10-HpOME	165530,8827	I	Ogliarola
ogliarola_infetti_213	10-HpOME	142789,317	I	Ogliarola
ogliarola_infetti_216	10-HpOME	18741,90967	I	Ogliarola
ogliarola_Ri_190	10-HpOME	26484,74541	R	Ogliarola
ogliarola_Ri_191	10-HpOME	41664,23943	R	Ogliarola
ogliarola_Ri_192	10-HpOME	31727,54524	R	Ogliarola
ogliarola_Ri_193	10-HpOME	18333,24064	R	Ogliarola
ogliarola_Ri_194	10-HpOME	58118,96653	R	Ogliarola
ogliarola_Ri_195	10-HpOME	70977,91072	R	Ogliarola
ogliarola_sana_154	10-HpOME	29714,73578	H	Ogliarola

ogliarola_sana_155	10-HpOME	32349,03585	H	Ogliarola
ogliarola_sana_156	10-HpOME	54802,23956	H	Ogliarola
ogliarola_sana_157	10-HpOME	61276,37059	H	Ogliarola
ogliarola_sana_158	10-HpOME	122335,8603	H	Ogliarola
ogliarola_sana_159	10-HpOME	112397,44	H	Ogliarola
cellina_infetti_200	11-HPODE	1270,936333	I	Cellina
cellina_infetti_201	11-HPODE	947,3327395	I	Cellina
cellina_infetti_208	11-HPODE	925,036916	I	Cellina
cellina_infetti_209	11-HPODE	978,7646061	I	Cellina
cellina_infetti_214	11-HPODE	1116,105692	I	Cellina
cellina_infetti_217	11-HPODE	1528,774826	I	Cellina
cellina_Ri_184	11-HPODE	3021,801174	R	Cellina
cellina_Ri_185	11-HPODE	3038,739588	R	Cellina
cellina_Ri_186	11-HPODE	2820,149612	R	Cellina
cellina_Ri_187	11-HPODE	2775,300231	R	Cellina
cellina_Ri_196	11-HPODE	4442,064851	R	Cellina
cellina_Ri_197	11-HPODE	2886,166941	R	Cellina
cellina_sani_169	11-HPODE	2445,72562	H	Cellina
cellina_sani_170	11-HPODE	3899,128564	H	Cellina
cellina_sani_171	11-HPODE	4505,150541	H	Cellina
cellina_sani_172	11-HPODE	7579,381461	H	Cellina
cellina_sani_176	11-HPODE	2050,263257	H	Cellina
cellina_sani_179	11-HPODE	2138,741766	H	Cellina
ogliarola_infetti_202	11-HPODE	2173,758884	I	Ogliarola
ogliarola_infetti_203	11-HPODE	3331,251531	I	Ogliarola
ogliarola_infetti_206	11-HPODE	2097,30973	I	Ogliarola
ogliarola_infetti_207	11-HPODE	10711,90531	I	Ogliarola
ogliarola_infetti_213	11-HPODE	12743,50149	I	Ogliarola
ogliarola_infetti_216	11-HPODE	1949,67017	I	Ogliarola
ogliarola_Ri_190	11-HPODE	1943,677547	R	Ogliarola
ogliarola_Ri_191	11-HPODE	2291,438664	R	Ogliarola
ogliarola_Ri_192	11-HPODE	1824,141555	R	Ogliarola
ogliarola_Ri_193	11-HPODE	1300,020638	R	Ogliarola
ogliarola_Ri_194	11-HPODE	2760,479122	R	Ogliarola
ogliarola_Ri_195	11-HPODE	5941,900067	R	Ogliarola
ogliarola_sana_154	11-HPODE	1951,407568	H	Ogliarola
ogliarola_sana_155	11-HPODE	1268,366766	H	Ogliarola
ogliarola_sana_156	11-HPODE	3363,318796	H	Ogliarola
ogliarola_sana_157	11-HPODE	4490,483572	H	Ogliarola
ogliarola_sana_158	11-HPODE	10160,36411	H	Ogliarola
ogliarola_sana_159	11-HPODE	10044,20283	H	Ogliarola
cellina_infetti_200	12,13-DiHOME	961,590337	I	Cellina
cellina_infetti_201	12,13-DiHOME	693,47596	I	Cellina
cellina_infetti_208	12,13-DiHOME	157,3818352	I	Cellina
cellina_infetti_209	12,13-DiHOME	132,243331	I	Cellina
cellina_infetti_214	12,13-DiHOME	304,6488732	I	Cellina

cellina_infetti_217	12,13-DiHOME	326,3674767	I	Cellina
cellina_Ri_184	12,13-DiHOME	503,1396501	R	Cellina
cellina_Ri_185	12,13-DiHOME	396,3778448	R	Cellina
cellina_Ri_186	12,13-DiHOME	364,5592581	R	Cellina
cellina_Ri_187	12,13-DiHOME	465,4440811	R	Cellina
cellina_Ri_196	12,13-DiHOME	2204,488996	R	Cellina
cellina_Ri_197	12,13-DiHOME	1026,163334	R	Cellina
cellina_sani_169	12,13-DiHOME	389,1303432	H	Cellina
cellina_sani_170	12,13-DiHOME	424,6208313	H	Cellina
cellina_sani_171	12,13-DiHOME	697,259385	H	Cellina
cellina_sani_172	12,13-DiHOME	1029,330259	H	Cellina
cellina_sani_176	12,13-DiHOME	655,5612159	H	Cellina
cellina_sani_179	12,13-DiHOME	520,5849122	H	Cellina
ogliarola_infetti_202	12,13-DiHOME	306,5215565	I	Ogliarola
ogliarola_infetti_203	12,13-DiHOME	429,8894206	I	Ogliarola
ogliarola_infetti_206	12,13-DiHOME	968,065932	I	Ogliarola
ogliarola_infetti_207	12,13-DiHOME	1928,289496	I	Ogliarola
ogliarola_infetti_213	12,13-DiHOME	2610,661131	I	Ogliarola
ogliarola_infetti_216	12,13-DiHOME	1123,719146	I	Ogliarola
ogliarola_Ri_190	12,13-DiHOME	149,3857208	R	Ogliarola
ogliarola_Ri_191	12,13-DiHOME	382,6418923	R	Ogliarola
ogliarola_Ri_192	12,13-DiHOME	264,4382343	R	Ogliarola
ogliarola_Ri_193	12,13-DiHOME	227,7992197	R	Ogliarola
ogliarola_Ri_194	12,13-DiHOME	1162,072806	R	Ogliarola
ogliarola_Ri_195	12,13-DiHOME	1378,611924	R	Ogliarola
ogliarola_sana_154	12,13-DiHOME	451,8307197	H	Ogliarola
ogliarola_sana_155	12,13-DiHOME	1054,536395	H	Ogliarola
ogliarola_sana_156	12,13-DiHOME	1037,773965	H	Ogliarola
ogliarola_sana_157	12,13-DiHOME	1337,037861	H	Ogliarola
ogliarola_sana_158	12,13-DiHOME	2417,515669	H	Ogliarola
ogliarola_sana_159	12,13-DiHOME	1495,347363	H	Ogliarola
cellina_infetti_200	12.13-EpOME	60220,27984	I	Cellina
cellina_infetti_201	12.13-EpOME	49465,1511	I	Cellina
cellina_infetti_208	12.13-EpOME	9448,696363	I	Cellina
cellina_infetti_209	12.13-EpOME	4513,024092	I	Cellina
cellina_infetti_214	12.13-EpOME	18047,90231	I	Cellina
cellina_infetti_217	12.13-EpOME	19279,27779	I	Cellina
cellina_Ri_184	12.13-EpOME	17930,14132	R	Cellina
cellina_Ri_185	12.13-EpOME	26308,90057	R	Cellina
cellina_Ri_186	12.13-EpOME	20727,78328	R	Cellina
cellina_Ri_187	12.13-EpOME	30726,27147	R	Cellina
cellina_Ri_196	12.13-EpOME	66907,23675	R	Cellina
cellina_Ri_197	12.13-EpOME	19043,25417	R	Cellina
cellina_sani_169	12.13-EpOME	25252,39516	H	Cellina
cellina_sani_170	12.13-EpOME	27607,917	H	Cellina
cellina_sani_171	12.13-EpOME	51453,36053	H	Cellina

cellina_sani_172	12.13-EpOME	55286,45003	H	Cellina
cellina_sani_176	12.13-EpOME	22067,79122	H	Cellina
cellina_sani_179	12.13-EpOME	43488,4711	H	Cellina
ogliarola_infetti_202	12.13-EpOME	23897,91611	I	Ogliarola
ogliarola_infetti_203	12.13-EpOME	42137,63642	I	Ogliarola
ogliarola_infetti_206	12.13-EpOME	32651,31668	I	Ogliarola
ogliarola_infetti_207	12.13-EpOME	88570,63144	I	Ogliarola
ogliarola_infetti_213	12.13-EpOME	238093,7923	I	Ogliarola
ogliarola_infetti_216	12.13-EpOME	17329,32494	I	Ogliarola
ogliarola_Ri_190	12.13-EpOME	5725,294989	R	Ogliarola
ogliarola_Ri_191	12.13-EpOME	23024,06792	R	Ogliarola
ogliarola_Ri_192	12.13-EpOME	17088,70868	R	Ogliarola
ogliarola_Ri_193	12.13-EpOME	13188,24807	R	Ogliarola
ogliarola_Ri_194	12.13-EpOME	30903,18405	R	Ogliarola
ogliarola_Ri_195	12.13-EpOME	63613,5836	R	Ogliarola
ogliarola_sana_154	12.13-EpOME	20711,94146	H	Ogliarola
ogliarola_sana_155	12.13-EpOME	14231,376	H	Ogliarola
ogliarola_sana_156	12.13-EpOME	19788,56797	H	Ogliarola
ogliarola_sana_157	12.13-EpOME	31570,90725	H	Ogliarola
ogliarola_sana_158	12.13-EpOME	81860,56717	H	Ogliarola
ogliarola_sana_159	12.13-EpOME	50950,77041	H	Ogliarola
cellina_infetti_200	13-HODE	21773,7453	I	Cellina
cellina_infetti_201	13-HODE	18252,8286	I	Cellina
cellina_infetti_208	13-HODE	3712,30936	I	Cellina
cellina_infetti_209	13-HODE	1690,30766	I	Cellina
cellina_infetti_214	13-HODE	6537,540263	I	Cellina
cellina_infetti_217	13-HODE	7011,678895	I	Cellina
cellina_Ri_184	13-HODE	7025,787649	R	Cellina
cellina_Ri_185	13-HODE	10163,60245	R	Cellina
cellina_Ri_186	13-HODE	8131,418257	R	Cellina
cellina_Ri_187	13-HODE	11880,80419	R	Cellina
cellina_Ri_196	13-HODE	25480,54283	R	Cellina
cellina_Ri_197	13-HODE	7291,639842	R	Cellina
cellina_sani_169	13-HODE	10148,9717	H	Cellina
cellina_sani_170	13-HODE	10828,9939	H	Cellina
cellina_sani_171	13-HODE	20258,83628	H	Cellina
cellina_sani_172	13-HODE	21766,42223	H	Cellina
cellina_sani_176	13-HODE	8156,169476	H	Cellina
cellina_sani_179	13-HODE	15591,32775	H	Cellina
ogliarola_infetti_202	13-HODE	9535,587155	I	Ogliarola
ogliarola_infetti_203	13-HODE	16509,28613	I	Ogliarola
ogliarola_infetti_206	13-HODE	15202,91614	I	Ogliarola
ogliarola_infetti_207	13-HODE	39250,73835	I	Ogliarola
ogliarola_infetti_213	13-HODE	90196,2272	I	Ogliarola
ogliarola_infetti_216	13-HODE	6412,480044	I	Ogliarola
ogliarola_Ri_190	13-HODE	2273,391706	R	Ogliarola

ogliarola_Ri_191	13-HODE	8986,254234	R	Ogliarola
ogliarola_Ri_192	13-HODE	6600,123985	R	Ogliarola
ogliarola_Ri_193	13-HODE	5479,972118	R	Ogliarola
ogliarola_Ri_194	13-HODE	12183,6161	R	Ogliarola
ogliarola_Ri_195	13-HODE	25037,37207	R	Ogliarola
ogliarola_sana_154	13-HODE	33747,59098	H	Ogliarola
ogliarola_sana_155	13-HODE	23300,07966	H	Ogliarola
ogliarola_sana_156	13-HODE	32952,18198	H	Ogliarola
ogliarola_sana_157	13-HODE	21005,56966	H	Ogliarola
ogliarola_sana_158	13-HODE	31657,9256	H	Ogliarola
ogliarola_sana_159	13-HODE	20476,2399	H	Ogliarola
cellina_infetti_200	13-HOTrE	6917,590464	I	Cellina
cellina_infetti_201	13-HOTrE	5444,996825	I	Cellina
cellina_infetti_208	13-HOTrE	966,3822542	I	Cellina
cellina_infetti_209	13-HOTrE	1089,038055	I	Cellina
cellina_infetti_214	13-HOTrE	2687,62102	I	Cellina
cellina_infetti_217	13-HOTrE	4086,579668	I	Cellina
cellina_Ri_184	13-HOTrE	2606,694945	R	Cellina
cellina_Ri_185	13-HOTrE	5805,175298	R	Cellina
cellina_Ri_186	13-HOTrE	4284,498645	R	Cellina
cellina_Ri_187	13-HOTrE	6656,020211	R	Cellina
cellina_Ri_196	13-HOTrE	6599,741048	R	Cellina
cellina_Ri_197	13-HOTrE	2401,436267	R	Cellina
cellina_sani_169	13-HOTrE	5655,20261	H	Cellina
cellina_sani_170	13-HOTrE	6153,739106	H	Cellina
cellina_sani_171	13-HOTrE	10120,65212	H	Cellina
cellina_sani_172	13-HOTrE	13808,97448	H	Cellina
cellina_sani_176	13-HOTrE	4451,100425	H	Cellina
cellina_sani_179	13-HOTrE	6788,761028	H	Cellina
ogliarola_infetti_202	13-HOTrE	6493,635747	I	Ogliarola
ogliarola_infetti_203	13-HOTrE	5138,351017	I	Ogliarola
ogliarola_infetti_206	13-HOTrE	6066,058791	I	Ogliarola
ogliarola_infetti_207	13-HOTrE	12190,82876	I	Ogliarola
ogliarola_infetti_213	13-HOTrE	31513,75072	I	Ogliarola
ogliarola_infetti_216	13-HOTrE	2968,580684	I	Ogliarola
ogliarola_Ri_190	13-HOTrE	2420,029267	R	Ogliarola
ogliarola_Ri_191	13-HOTrE	5956,334045	R	Ogliarola
ogliarola_Ri_192	13-HOTrE	4683,652886	R	Ogliarola
ogliarola_Ri_193	13-HOTrE	4584,350116	R	Ogliarola
ogliarola_Ri_194	13-HOTrE	6577,677806	R	Ogliarola
ogliarola_Ri_195	13-HOTrE	17149,54279	R	Ogliarola
ogliarola_sana_154	13-HOTrE	4435,35073	H	Ogliarola
ogliarola_sana_155	13-HOTrE	7716,673595	H	Ogliarola
ogliarola_sana_156	13-HOTrE	7304,532402	H	Ogliarola
ogliarola_sana_157	13-HOTrE	8128,431919	H	Ogliarola
ogliarola_sana_158	13-HOTrE	14532,12375	H	Ogliarola

ogliarola_sana_159	13-HOTrE	8832,491758	H	Ogliarola
cellina_infetti_200	13-HpODE	343,130118	I	Cellina
cellina_infetti_201	13-HpODE	365,6438407	I	Cellina
cellina_infetti_208	13-HpODE	276,7993292	I	Cellina
cellina_infetti_209	13-HpODE	155,7344353	I	Cellina
cellina_infetti_214	13-HpODE	471,1469498	I	Cellina
cellina_infetti_217	13-HpODE	168,5449035	I	Cellina
cellina_Ri_184	13-HpODE	915,7096146	R	Cellina
cellina_Ri_185	13-HpODE	1005,716953	R	Cellina
cellina_Ri_186	13-HpODE	1320,59128	R	Cellina
cellina_Ri_187	13-HpODE	1292,71993	R	Cellina
cellina_Ri_196	13-HpODE	381,4007859	R	Cellina
cellina_Ri_197	13-HpODE	265,0006128	R	Cellina
cellina_sani_169	13-HpODE	2495,396337	H	Cellina
cellina_sani_170	13-HpODE	1830,49358	H	Cellina
cellina_sani_171	13-HpODE	5077,436698	H	Cellina
cellina_sani_172	13-HpODE	5030,343221	H	Cellina
cellina_sani_176	13-HpODE	513,2947857	H	Cellina
cellina_sani_179	13-HpODE	3164,097757	H	Cellina
ogliarola_infetti_202	13-HpODE	1403,211772	I	Ogliarola
ogliarola_infetti_203	13-HpODE	2192,349011	I	Ogliarola
ogliarola_infetti_206	13-HpODE	399,3553384	I	Ogliarola
ogliarola_infetti_207	13-HpODE	2462,969009	I	Ogliarola
ogliarola_infetti_213	13-HpODE	5327,675774	I	Ogliarola
ogliarola_infetti_216	13-HpODE	356,7832351	I	Ogliarola
ogliarola_Ri_190	13-HpODE	381,6712347	R	Ogliarola
ogliarola_Ri_191	13-HpODE	956,841698	R	Ogliarola
ogliarola_Ri_192	13-HpODE	526,8537147	R	Ogliarola
ogliarola_Ri_193	13-HpODE	572,3365862	R	Ogliarola
ogliarola_Ri_194	13-HpODE	464,9051456	R	Ogliarola
ogliarola_Ri_195	13-HpODE	1645,60694	R	Ogliarola
ogliarola_sana_154	13-HpODE	2066,560246	H	Ogliarola
ogliarola_sana_155	13-HpODE	839,94589	H	Ogliarola
ogliarola_sana_156	13-HpODE	1456,519598	H	Ogliarola
ogliarola_sana_157	13-HpODE	1356,958731	H	Ogliarola
ogliarola_sana_158	13-HpODE	2028,875176	H	Ogliarola
ogliarola_sana_159	13-HpODE	1057,388492	H	Ogliarola
cellina_infetti_200	13-HpOTrE	542,5362441	I	Cellina
cellina_infetti_201	13-HpOTrE	410,2861079	I	Cellina
cellina_infetti_208	13-HpOTrE	125,0272097	I	Cellina
cellina_infetti_209	13-HpOTrE	111,6669678	I	Cellina
cellina_infetti_214	13-HpOTrE	323,8462503	I	Cellina
cellina_infetti_217	13-HpOTrE	496,1875113	I	Cellina
cellina_Ri_184	13-HpOTrE	308,2184877	R	Cellina
cellina_Ri_185	13-HpOTrE	518,1882482	R	Cellina
cellina_Ri_186	13-HpOTrE	491,6058872	R	Cellina

cellina_Ri_187	13-HpOTrE	845,7433197	R	Cellina
cellina_Ri_196	13-HpOTrE	1059,704578	R	Cellina
cellina_Ri_197	13-HpOTrE	333,2797507	R	Cellina
cellina_sani_169	13-HpOTrE	1703,60454	H	Cellina
cellina_sani_170	13-HpOTrE	1951,910653	H	Cellina
cellina_sani_171	13-HpOTrE	2426,321164	H	Cellina
cellina_sani_172	13-HpOTrE	2983,080902	H	Cellina
cellina_sani_176	13-HpOTrE	1053,38238	H	Cellina
cellina_sani_179	13-HpOTrE	1903,852703	H	Cellina
ogliarola_infetti_202	13-HpOTrE	452,2749242	I	Ogliarola
ogliarola_infetti_203	13-HpOTrE	332,1315924	I	Ogliarola
ogliarola_infetti_206	13-HpOTrE	596,9679363	I	Ogliarola
ogliarola_infetti_207	13-HpOTrE	1081,971957	I	Ogliarola
ogliarola_infetti_213	13-HpOTrE	3640,113179	I	Ogliarola
ogliarola_infetti_216	13-HpOTrE	537,1013266	I	Ogliarola
ogliarola_Ri_190	13-HpOTrE	269,4223362	R	Ogliarola
ogliarola_Ri_191	13-HpOTrE	585,8011831	R	Ogliarola
ogliarola_Ri_192	13-HpOTrE	572,0589766	R	Ogliarola
ogliarola_Ri_193	13-HpOTrE	410,9588611	R	Ogliarola
ogliarola_Ri_194	13-HpOTrE	1067,334244	R	Ogliarola
ogliarola_Ri_195	13-HpOTrE	3374,141684	R	Ogliarola
ogliarola_sana_154	13-HpOTrE	796,3633849	H	Ogliarola
ogliarola_sana_155	13-HpOTrE	3586,366719	H	Ogliarola
ogliarola_sana_156	13-HpOTrE	2312,218768	H	Ogliarola
ogliarola_sana_157	13-HpOTrE	2858,470671	H	Ogliarola
ogliarola_sana_158	13-HpOTrE	2555,803992	H	Ogliarola
ogliarola_sana_159	13-HpOTrE	2550,872535	H	Ogliarola
cellina_infetti_200	13-oxoODE	4480,998089	I	Cellina
cellina_infetti_201	13-oxoODE	4232,864768	I	Cellina
cellina_infetti_208	13-oxoODE	1453,801799	I	Cellina
cellina_infetti_209	13-oxoODE	800,7562374	I	Cellina
cellina_infetti_214	13-oxoODE	2347,91754	I	Cellina
cellina_infetti_217	13-oxoODE	2051,237352	I	Cellina
cellina_Ri_184	13-oxoODE	3695,07534	R	Cellina
cellina_Ri_185	13-oxoODE	4851,792379	R	Cellina
cellina_Ri_186	13-oxoODE	4614,700333	R	Cellina
cellina_Ri_187	13-oxoODE	5037,671701	R	Cellina
cellina_Ri_196	13-oxoODE	17253,89737	R	Cellina
cellina_Ri_197	13-oxoODE	3259,735978	R	Cellina
cellina_sani_169	13-oxoODE	6523,539307	H	Cellina
cellina_sani_170	13-oxoODE	5656,100294	H	Cellina
cellina_sani_171	13-oxoODE	13552,92042	H	Cellina
cellina_sani_172	13-oxoODE	13598,65092	H	Cellina
cellina_sani_176	13-oxoODE	3038,277342	H	Cellina
cellina_sani_179	13-oxoODE	6805,303867	H	Cellina
ogliarola_infetti_202	13-oxoODE	4699,478431	I	Ogliarola

ogliarola_infetti_203	13-oxoODE	6786,727948	I	Ogliarola
ogliarola_infetti_206	13-oxoODE	5553,331652	I	Ogliarola
ogliarola_infetti_207	13-oxoODE	17391,87719	I	Ogliarola
ogliarola_infetti_213	13-oxoODE	37425,71317	I	Ogliarola
ogliarola_infetti_216	13-oxoODE	2090,497057	I	Ogliarola
ogliarola_Ri_190	13-oxoODE	824,6023976	R	Ogliarola
ogliarola_Ri_191	13-oxoODE	4638,006119	R	Ogliarola
ogliarola_Ri_192	13-oxoODE	3065,745248	R	Ogliarola
ogliarola_Ri_193	13-oxoODE	2312,468326	R	Ogliarola
ogliarola_Ri_194	13-oxoODE	4133,589832	R	Ogliarola
ogliarola_Ri_195	13-oxoODE	10480,47712	R	Ogliarola
ogliarola_sana_154	13-oxoODE	7377,146384	H	Ogliarola
ogliarola_sana_155	13-oxoODE	3780,310585	H	Ogliarola
ogliarola_sana_156	13-oxoODE	4641,117893	H	Ogliarola
ogliarola_sana_157	13-oxoODE	4917,931337	H	Ogliarola
ogliarola_sana_158	13-oxoODE	11428,40741	H	Ogliarola
ogliarola_sana_159	13-oxoODE	6162,958006	H	Ogliarola
cellina_infetti_200	JA	893,8742129	I	Cellina
cellina_infetti_201	JA	662,6438074	I	Cellina
cellina_infetti_208	JA	252,3860112	I	Cellina
cellina_infetti_209	JA	330,9725495	I	Cellina
cellina_infetti_214	JA	625,7460945	I	Cellina
cellina_infetti_217	JA	735,0030404	I	Cellina
cellina_Ri_184	JA	422,9973966	R	Cellina
cellina_Ri_185	JA	492,1255334	R	Cellina
cellina_Ri_186	JA	415,5825253	R	Cellina
cellina_Ri_187	JA	320,715725	R	Cellina
cellina_Ri_196	JA	2071,805293	R	Cellina
cellina_Ri_197	JA	1100,997842	R	Cellina
cellina_sani_169	JA	255,0703831	H	Cellina
cellina_sani_170	JA	495,8021326	H	Cellina
cellina_sani_171	JA	338,9682702	H	Cellina
cellina_sani_172	JA	386,8672739	H	Cellina
cellina_sani_176	JA	343,491212	H	Cellina
cellina_sani_179	JA	717,2856691	H	Cellina
ogliarola_infetti_202	JA	217,5154479	I	Ogliarola
ogliarola_infetti_203	JA	271,4008319	I	Ogliarola
ogliarola_infetti_206	JA	1503,737786	I	Ogliarola
ogliarola_infetti_207	JA	2232,174532	I	Ogliarola
ogliarola_infetti_213	JA	4015,423586	I	Ogliarola
ogliarola_infetti_216	JA	318,2835303	I	Ogliarola
ogliarola_Ri_190	JA	1234,448207	R	Ogliarola
ogliarola_Ri_191	JA	610,9984247	R	Ogliarola
ogliarola_Ri_192	JA	361,1206378	R	Ogliarola
ogliarola_Ri_193	JA	299,8008395	R	Ogliarola
ogliarola_Ri_194	JA	3129,735218	R	Ogliarola

ogliarola_Ri_195	JA	2702,156118	R	Ogliarola
ogliarola_sana_154	JA	185,237846	H	Ogliarola
ogliarola_sana_155	JA	266,7392753	H	Ogliarola
ogliarola_sana_156	JA	361,7236142	H	Ogliarola
ogliarola_sana_157	JA	302,1043665	H	Ogliarola
ogliarola_sana_158	JA	1399,155527	H	Ogliarola
ogliarola_sana_159	JA	720,1509294	H	Ogliarola
cellina_infetti_200	8,13-diHODE	23857,4964	I	Cellina
cellina_infetti_201	8,13-diHODE	32947,55994	I	Cellina
cellina_infetti_208	8,13-diHODE	6280,585266	I	Cellina
cellina_infetti_209	8,13-diHODE	2804,34635	I	Cellina
cellina_infetti_214	8,13-diHODE	14844,51755	I	Cellina
cellina_infetti_217	8,13-diHODE	13569,22016	I	Cellina
cellina_Ri_184	8,13-diHODE	7500,776956	R	Cellina
cellina_Ri_185	8,13-diHODE	9385,84852	R	Cellina
cellina_Ri_186	8,13-diHODE	10878,93606	R	Cellina
cellina_Ri_187	8,13-diHODE	14946,48116	R	Cellina
cellina_Ri_196	8,13-diHODE	37075,85797	R	Cellina
cellina_Ri_197	8,13-diHODE	10768,89721	R	Cellina
cellina_sani_169	8,13-diHODE	15666,14437	H	Cellina
cellina_sani_170	8,13-diHODE	12158,36667	H	Cellina
cellina_sani_171	8,13-diHODE	36884,61599	H	Cellina
cellina_sani_172	8,13-diHODE	37267,38606	H	Cellina
cellina_sani_176	8,13-diHODE	19724,85738	H	Cellina
cellina_sani_179	8,13-diHODE	21974,8865	H	Cellina
ogliarola_infetti_202	8,13-diHODE	8205,069844	I	Ogliarola
ogliarola_infetti_203	8,13-diHODE	14565,54295	I	Ogliarola
ogliarola_infetti_206	8,13-diHODE	24294,92014	I	Ogliarola
ogliarola_infetti_207	8,13-diHODE	65614,16136	I	Ogliarola
ogliarola_infetti_213	8,13-diHODE	167828,009	I	Ogliarola
ogliarola_infetti_216	8,13-diHODE	12878,08955	I	Ogliarola
ogliarola_Ri_190	8,13-diHODE	2561,890793	R	Ogliarola
ogliarola_Ri_191	8,13-diHODE	7959,65554	R	Ogliarola
ogliarola_Ri_192	8,13-diHODE	7714,520418	R	Ogliarola
ogliarola_Ri_193	8,13-diHODE	4671,711549	R	Ogliarola
ogliarola_Ri_194	8,13-diHODE	9222,907213	R	Ogliarola
ogliarola_Ri_195	8,13-diHODE	22600,37434	R	Ogliarola
ogliarola_sana_154	8,13-diHODE	15275,25096	H	Ogliarola
ogliarola_sana_155	8,13-diHODE	18658,57299	H	Ogliarola
ogliarola_sana_156	8,13-diHODE	15578,73352	H	Ogliarola
ogliarola_sana_157	8,13-diHODE	16025,28018	H	Ogliarola
ogliarola_sana_158	8,13-diHODE	7269,41752	H	Ogliarola
ogliarola_sana_159	8,13-diHODE	6051,261492	H	Ogliarola
cellina_infetti_200	8-HODE	176,2085919	I	Cellina
cellina_infetti_201	8-HODE	221,7687074	I	Cellina
cellina_infetti_208	8-HODE	49,25553835	I	Cellina

cellina_infetti_209	8-HODE	50,93060379	I	Cellina
cellina_infetti_214	8-HODE	231,6727931	I	Cellina
cellina_infetti_217	8-HODE	170,9926051	I	Cellina
cellina_Ri_184	8-HODE	645,7642847	R	Cellina
cellina_Ri_185	8-HODE	185,996792	R	Cellina
cellina_Ri_186	8-HODE	377,2235273	R	Cellina
cellina_Ri_187	8-HODE	210,9081522	R	Cellina
cellina_Ri_196	8-HODE	848,0590639	R	Cellina
cellina_Ri_197	8-HODE	395,7480925	R	Cellina
cellina_sani_169	8-HODE	498,81033	H	Cellina
cellina_sani_170	8-HODE	404,9766677	H	Cellina
cellina_sani_171	8-HODE	547,9334333	H	Cellina
cellina_sani_172	8-HODE	670,2953323	H	Cellina
cellina_sani_176	8-HODE	452,6290606	H	Cellina
cellina_sani_179	8-HODE	527,6526209	H	Cellina
ogliarola_infetti_202	8-HODE	126,8726889	I	Ogliarola
ogliarola_infetti_203	8-HODE	136,5881978	I	Ogliarola
ogliarola_infetti_206	8-HODE	204,8587494	I	Ogliarola
ogliarola_infetti_207	8-HODE	744,6367525	I	Ogliarola
ogliarola_infetti_213	8-HODE	1422,220569	I	Ogliarola
ogliarola_infetti_216	8-HODE	127,5713939	I	Ogliarola
ogliarola_Ri_190	8-HODE	96,34710148	R	Ogliarola
ogliarola_Ri_191	8-HODE	207,8960507	R	Ogliarola
ogliarola_Ri_192	8-HODE	89,09122301	R	Ogliarola
ogliarola_Ri_193	8-HODE	69,0657701	R	Ogliarola
ogliarola_Ri_194	8-HODE	331,2023775	R	Ogliarola
ogliarola_Ri_195	8-HODE	582,6283904	R	Ogliarola
ogliarola_sana_154	8-HODE	539,8923058	H	Ogliarola
ogliarola_sana_155	8-HODE	166,3017304	H	Ogliarola
ogliarola_sana_156	8-HODE	552,7667641	H	Ogliarola
ogliarola_sana_157	8-HODE	507,6252405	H	Ogliarola
ogliarola_sana_158	8-HODE	717,8946205	H	Ogliarola
ogliarola_sana_159	8-HODE	839,1583729	H	Ogliarola
cellina_infetti_200	8-HpODE	21860,53085	I	Cellina
cellina_infetti_201	8-HpODE	30266,79271	I	Cellina
cellina_infetti_208	8-HpODE	5771,165374	I	Cellina
cellina_infetti_209	8-HpODE	2370,764633	I	Cellina
cellina_infetti_214	8-HpODE	14052,9179	I	Cellina
cellina_infetti_217	8-HpODE	12691,08799	I	Cellina
cellina_Ri_184	8-HpODE	7057,471581	R	Cellina
cellina_Ri_185	8-HpODE	8661,879438	R	Cellina
cellina_Ri_186	8-HpODE	9973,296215	R	Cellina
cellina_Ri_187	8-HpODE	14576,53971	R	Cellina
cellina_Ri_196	8-HpODE	35579,769	R	Cellina
cellina_Ri_197	8-HpODE	10495,29159	R	Cellina
cellina_sani_169	8-HpODE	14744,1292	H	Cellina

cellina_sani_170	8-HpODE	11316,13112	H	Cellina
cellina_sani_171	8-HpODE	34925,02418	H	Cellina
cellina_sani_172	8-HpODE	34459,55454	H	Cellina
cellina_sani_176	8-HpODE	18046,76281	H	Cellina
cellina_sani_179	8-HpODE	20291,40079	H	Cellina
ogliarola_infetti_202	8-HpODE	7840,261425	I	Ogliarola
ogliarola_infetti_203	8-HpODE	13430,53024	I	Ogliarola
ogliarola_infetti_206	8-HpODE	23624,03487	I	Ogliarola
ogliarola_infetti_207	8-HpODE	59819,38836	I	Ogliarola
ogliarola_infetti_213	8-HpODE	165689,0382	I	Ogliarola
ogliarola_infetti_216	8-HpODE	11948,2578	I	Ogliarola
ogliarola_Ri_190	8-HpODE	2493,18122	R	Ogliarola
ogliarola_Ri_191	8-HpODE	6985,519527	R	Ogliarola
ogliarola_Ri_192	8-HpODE	7450,528962	R	Ogliarola
ogliarola_Ri_193	8-HpODE	4358,179815	R	Ogliarola
ogliarola_Ri_194	8-HpODE	9129,143165	R	Ogliarola
ogliarola_Ri_195	8-HpODE	22456,52514	R	Ogliarola
ogliarola_sana_154	8-HpODE	13382,8428	H	Ogliarola
ogliarola_sana_155	8-HpODE	16843,6035	H	Ogliarola
ogliarola_sana_156	8-HpODE	14478,73477	H	Ogliarola
ogliarola_sana_157	8-HpODE	14591,31727	H	Ogliarola
ogliarola_sana_158	8-HpODE	9297,67713	H	Ogliarola
ogliarola_sana_159	8-HpODE	7256,43238	H	Ogliarola
cellina_infetti_200	9,10-EpOME	64113,50983	I	Cellina
cellina_infetti_201	9,10-EpOME	60664,53551	I	Cellina
cellina_infetti_208	9,10-EpOME	12243,4045	I	Cellina
cellina_infetti_209	9,10-EpOME	5542,622045	I	Cellina
cellina_infetti_214	9,10-EpOME	21762,60497	I	Cellina
cellina_infetti_217	9,10-EpOME	21060,97897	I	Cellina
cellina_Ri_184	9,10-EpOME	16255,03468	R	Cellina
cellina_Ri_185	9,10-EpOME	26536,77053	R	Cellina
cellina_Ri_186	9,10-EpOME	18307,06137	R	Cellina
cellina_Ri_187	9,10-EpOME	26695,67724	R	Cellina
cellina_Ri_196	9,10-EpOME	70017,05964	R	Cellina
cellina_Ri_197	9,10-EpOME	19989,42571	R	Cellina
cellina_sani_169	9,10-EpOME	22266,78943	H	Cellina
cellina_sani_170	9,10-EpOME	21366,90248	H	Cellina
cellina_sani_171	9,10-EpOME	50311,89243	H	Cellina
cellina_sani_172	9,10-EpOME	54484,39031	H	Cellina
cellina_sani_176	9,10-EpOME	18792,33442	H	Cellina
cellina_sani_179	9,10-EpOME	29793,39089	H	Cellina
ogliarola_infetti_202	9,10-EpOME	22630,71176	I	Ogliarola
ogliarola_infetti_203	9,10-EpOME	45015,73919	I	Ogliarola
ogliarola_infetti_206	9,10-EpOME	33460,71663	I	Ogliarola
ogliarola_infetti_207	9,10-EpOME	131422,9246	I	Ogliarola
ogliarola_infetti_213	9,10-EpOME	334578,5892	I	Ogliarola

ogliarola_infetti_216	9,10-EpOME	17852,55362	I	Ogliarola
ogliarola_Ri_190	9,10-EpOME	5373,24868	R	Ogliarola
ogliarola_Ri_191	9,10-EpOME	20872,46119	R	Ogliarola
ogliarola_Ri_192	9,10-EpOME	12124,07872	R	Ogliarola
ogliarola_Ri_193	9,10-EpOME	13348,75649	R	Ogliarola
ogliarola_Ri_194	9,10-EpOME	23670,82772	R	Ogliarola
ogliarola_Ri_195	9,10-EpOME	46408,95599	R	Ogliarola
ogliarola_sana_154	9,10-EpOME	49651,58945	H	Ogliarola
ogliarola_sana_155	9,10-EpOME	44383,73017	H	Ogliarola
ogliarola_sana_156	9,10-EpOME	46838,56918	H	Ogliarola
ogliarola_sana_157	9,10-EpOME	34071,5937	H	Ogliarola
ogliarola_sana_158	9,10-EpOME	29338,39065	H	Ogliarola
ogliarola_sana_159	9,10-EpOME	43943,40537	H	Ogliarola
cellina_infetti_200	9-HODE	114990,4147	I	Cellina
cellina_infetti_201	9-HODE	109655,7445	I	Cellina
cellina_infetti_208	9-HODE	22464,05916	I	Cellina
cellina_infetti_209	9-HODE	9954,421786	I	Cellina
cellina_infetti_214	9-HODE	39201,63373	I	Cellina
cellina_infetti_217	9-HODE	37946,5056	I	Cellina
cellina_Ri_184	9-HODE	29344,56983	R	Cellina
cellina_Ri_185	9-HODE	48255,85774	R	Cellina
cellina_Ri_186	9-HODE	33253,06199	R	Cellina
cellina_Ri_187	9-HODE	48442,22013	R	Cellina
cellina_Ri_196	9-HODE	117796,256	R	Cellina
cellina_Ri_197	9-HODE	34425,48257	R	Cellina
cellina_sani_169	9-HODE	40358,13001	H	Cellina
cellina_sani_170	9-HODE	38131,00128	H	Cellina
cellina_sani_171	9-HODE	91110,54274	H	Cellina
cellina_sani_172	9-HODE	98329,25483	H	Cellina
cellina_sani_176	9-HODE	34350,09675	H	Cellina
cellina_sani_179	9-HODE	53856,16797	H	Cellina
ogliarola_infetti_202	9-HODE	41021,1649	I	Ogliarola
ogliarola_infetti_203	9-HODE	81766,37346	I	Ogliarola
ogliarola_infetti_206	9-HODE	61534,42511	I	Ogliarola
ogliarola_infetti_207	9-HODE	239991,9842	I	Ogliarola
ogliarola_infetti_213	9-HODE	607109,1606	I	Ogliarola
ogliarola_infetti_216	9-HODE	32225,1482	I	Ogliarola
ogliarola_Ri_190	9-HODE	9403,727424	R	Ogliarola
ogliarola_Ri_191	9-HODE	37015,57699	R	Ogliarola
ogliarola_Ri_192	9-HODE	21248,26664	R	Ogliarola
ogliarola_Ri_193	9-HODE	24217,90964	R	Ogliarola
ogliarola_Ri_194	9-HODE	31820,10427	R	Ogliarola
ogliarola_Ri_195	9-HODE	82680,96638	R	Ogliarola
ogliarola_sana_154	9-HODE	49651,58945	H	Ogliarola
ogliarola_sana_155	9-HODE	44383,73017	H	Ogliarola
ogliarola_sana_156	9-HODE	46838,56918	H	Ogliarola

ogliarola_sana_157	9-HODE	49944,5816	H	Ogliarola
ogliarola_sana_158	9-HODE	48716,27955	H	Ogliarola
ogliarola_sana_159	9-HODE	66800,22719	H	Ogliarola
cellina_infetti_200	9-HOTrE	16879,43889	I	Cellina
cellina_infetti_201	9-HOTrE	14991,51921	I	Cellina
cellina_infetti_208	9-HOTrE	2940,539952	I	Cellina
cellina_infetti_209	9-HOTrE	2927,34893	I	Cellina
cellina_infetti_214	9-HOTrE	8690,933021	I	Cellina
cellina_infetti_217	9-HOTrE	11359,95596	I	Cellina
cellina_Ri_184	9-HOTrE	5227,887022	R	Cellina
cellina_Ri_185	9-HOTrE	12182,03757	R	Cellina
cellina_Ri_186	9-HOTrE	7906,705956	R	Cellina
cellina_Ri_187	9-HOTrE	13813,43722	R	Cellina
cellina_Ri_196	9-HOTrE	20339,22784	R	Cellina
cellina_Ri_197	9-HOTrE	6931,510052	R	Cellina
cellina_sani_169	9-HOTrE	15440,8299	H	Cellina
cellina_sani_170	9-HOTrE	16264,65528	H	Cellina
cellina_sani_171	9-HOTrE	30816,41043	H	Cellina
cellina_sani_172	9-HOTrE	43463,14839	H	Cellina
cellina_sani_176	9-HOTrE	11170,21961	H	Cellina
cellina_sani_179	9-HOTrE	14715,49741	H	Cellina
ogliarola_infetti_202	9-HOTrE	13014,30661	I	Ogliarola
ogliarola_infetti_203	9-HOTrE	12488,78009	I	Ogliarola
ogliarola_infetti_206	9-HOTrE	13262,0492	I	Ogliarola
ogliarola_infetti_207	9-HOTrE	38496,19243	I	Ogliarola
ogliarola_infetti_213	9-HOTrE	95601,65488	I	Ogliarola
ogliarola_infetti_216	9-HOTrE	8859,645776	I	Ogliarola
ogliarola_Ri_190	9-HOTrE	5897,909083	R	Ogliarola
ogliarola_Ri_191	9-HOTrE	10381,6921	R	Ogliarola
ogliarola_Ri_192	9-HOTrE	7943,934942	R	Ogliarola
ogliarola_Ri_193	9-HOTrE	9213,715328	R	Ogliarola
ogliarola_Ri_194	9-HOTrE	14895,77741	R	Ogliarola
ogliarola_Ri_195	9-HOTrE	30094,78728	R	Ogliarola
ogliarola_sana_154	9-HOTrE	14601,77442	H	Ogliarola
ogliarola_sana_155	9-HOTrE	18886,76936	H	Ogliarola
ogliarola_sana_156	9-HOTrE	17297,69938	H	Ogliarola
ogliarola_sana_157	9-HOTrE	18247,3914	H	Ogliarola
ogliarola_sana_158	9-HOTrE	17189,24428	H	Ogliarola
ogliarola_sana_159	9-HOTrE	25298,40478	H	Ogliarola
cellina_infetti_200	9-HpODE	300,1660007	I	Cellina
cellina_infetti_201	9-HpODE	96,17698751	I	Cellina
cellina_infetti_208	9-HpODE	816,4892612	I	Cellina
cellina_infetti_209	9-HpODE	384,4877288	I	Cellina
cellina_infetti_214	9-HpODE	320,4254517	I	Cellina
cellina_infetti_217	9-HpODE	70,21839933	I	Cellina
cellina_Ri_184	9-HpODE	1257,604607	R	Cellina

cellina_Ri_185	9-HpODE	1538,205418	R	Cellina
cellina_Ri_186	9-HpODE	2254,558249	R	Cellina
cellina_Ri_187	9-HpODE	2519,571905	R	Cellina
cellina_Ri_196	9-HpODE	1136,490632	R	Cellina
cellina_Ri_197	9-HpODE	823,4010428	R	Cellina
cellina_sani_169	9-HpODE	3296,867932	H	Cellina
cellina_sani_170	9-HpODE	2421,330216	H	Cellina
cellina_sani_171	9-HpODE	8741,072703	H	Cellina
cellina_sani_172	9-HpODE	9141,139035	H	Cellina
cellina_sani_176	9-HpODE	415,4258196	H	Cellina
cellina_sani_179	9-HpODE	2213,082772	H	Cellina
ogliarola_infetti_202	9-HpODE	2435,660671	I	Ogliarola
ogliarola_infetti_203	9-HpODE	5051,796287	I	Ogliarola
ogliarola_infetti_206	9-HpODE	288,2204137	I	Ogliarola
ogliarola_infetti_207	9-HpODE	5965,549262	I	Ogliarola
ogliarola_infetti_213	9-HpODE	13098,93982	I	Ogliarola
ogliarola_infetti_216	9-HpODE	225,7299214	I	Ogliarola
ogliarola_Ri_190	9-HpODE	203,6781714	R	Ogliarola
ogliarola_Ri_191	9-HpODE	1287,445282	R	Ogliarola
ogliarola_Ri_192	9-HpODE	695,685505	R	Ogliarola
ogliarola_Ri_193	9-HpODE	759,5904745	R	Ogliarola
ogliarola_Ri_194	9-HpODE	548,4211659	R	Ogliarola
ogliarola_Ri_195	9-HpODE	2541,734129	R	Ogliarola
ogliarola_sana_154	9-HpODE	2591,030746	H	Ogliarola
ogliarola_sana_155	9-HpODE	922,3013145	H	Ogliarola
ogliarola_sana_156	9-HpODE	1494,155318	H	Ogliarola
ogliarola_sana_157	9-HpODE	1381,039786	H	Ogliarola
ogliarola_sana_158	9-HpODE	2534,395333	H	Ogliarola
ogliarola_sana_159	9-HpODE	1150,938968	H	Ogliarola
cellina_infetti_200	9-HpOTre	608,5432728	I	Cellina
cellina_infetti_201	9-HpOTre	713,3103712	I	Cellina
cellina_infetti_208	9-HpOTre	571,7648093	I	Cellina
cellina_infetti_209	9-HpOTre	606,079841	I	Cellina
cellina_infetti_214	9-HpOTre	559,415363	I	Cellina
cellina_infetti_217	9-HpOTre	319,344306	I	Cellina
cellina_Ri_184	9-HpOTre	1124,438102	R	Cellina
cellina_Ri_185	9-HpOTre	1900,635782	R	Cellina
cellina_Ri_186	9-HpOTre	2132,674535	R	Cellina
cellina_Ri_187	9-HpOTre	2575,800542	R	Cellina
cellina_Ri_196	9-HpOTre	2008,055747	R	Cellina
cellina_Ri_197	9-HpOTre	677,0394051	R	Cellina
cellina_sani_169	9-HpOTre	3957,475506	H	Cellina
cellina_sani_170	9-HpOTre	3944,714192	H	Cellina
cellina_sani_171	9-HpOTre	10953,19335	H	Cellina
cellina_sani_172	9-HpOTre	14419,97509	H	Cellina
cellina_sani_176	9-HpOTre	917,399829	H	Cellina

cellina_sani_179	9-HpOTre	1932,051625	H	Cellina
ogliarola_infetti_202	9-HpOTre	2833,348834	I	Ogliarola
ogliarola_infetti_203	9-HpOTre	2362,880045	I	Ogliarola
ogliarola_infetti_206	9-HpOTre	737,2993476	I	Ogliarola
ogliarola_infetti_207	9-HpOTre	9146,981831	I	Ogliarola
ogliarola_infetti_213	9-HpOTre	15995,22221	I	Ogliarola
ogliarola_infetti_216	9-HpOTre	442,0167755	I	Ogliarola
ogliarola_Ri_190	9-HpOTre	255,1566594	R	Ogliarola
ogliarola_Ri_191	9-HpOTre	1701,793577	R	Ogliarola
ogliarola_Ri_192	9-HpOTre	1209,460182	R	Ogliarola
ogliarola_Ri_193	9-HpOTre	1126,034287	R	Ogliarola
ogliarola_Ri_194	9-HpOTre	1361,68343	R	Ogliarola
ogliarola_Ri_195	9-HpOTre	4332,151197	R	Ogliarola
ogliarola_sana_154	9-HpOTre	1464,886516	H	Ogliarola
ogliarola_sana_155	9-HpOTre	906,9727025	H	Ogliarola
ogliarola_sana_156	9-HpOTre	1288,851356	H	Ogliarola
ogliarola_sana_157	9-HpOTre	1369,112869	H	Ogliarola
ogliarola_sana_158	9-HpOTre	7690,583528	H	Ogliarola
ogliarola_sana_159	9-HpOTre	2767,169339	H	Ogliarola
cellina_infetti_200	9-oxoODE	7085,938901	I	Cellina
cellina_infetti_201	9-oxoODE	8128,75717	I	Cellina
cellina_infetti_208	9-oxoODE	3213,840194	I	Cellina
cellina_infetti_209	9-oxoODE	1446,266339	I	Cellina
cellina_infetti_214	9-oxoODE	2819,568962	I	Cellina
cellina_infetti_217	9-oxoODE	2547,745892	I	Cellina
cellina_Ri_184	9-oxoODE	3404,456388	R	Cellina
cellina_Ri_185	9-oxoODE	5746,35348	R	Cellina
cellina_Ri_186	9-oxoODE	4699,995147	R	Cellina
cellina_Ri_187	9-oxoODE	6416,828545	R	Cellina
cellina_Ri_196	9-oxoODE	16006,56978	R	Cellina
cellina_Ri_197	9-oxoODE	3705,172088	R	Cellina
cellina_sani_169	9-oxoODE	6820,728551	H	Cellina
cellina_sani_170	9-oxoODE	5758,742537	H	Cellina
cellina_sani_171	9-oxoODE	16714,46892	H	Cellina
cellina_sani_172	9-oxoODE	18395,03302	H	Cellina
cellina_sani_176	9-oxoODE	3116,184172	H	Cellina
cellina_sani_179	9-oxoODE	2694,78565	H	Cellina
ogliarola_infetti_202	9-oxoODE	6406,200362	I	Ogliarola
ogliarola_infetti_203	9-oxoODE	12481,5208	I	Ogliarola
ogliarola_infetti_206	9-oxoODE	6195,437432	I	Ogliarola
ogliarola_infetti_207	9-oxoODE	31473,88568	I	Ogliarola
ogliarola_infetti_213	9-oxoODE	56307,73745	I	Ogliarola
ogliarola_infetti_216	9-oxoODE	2421,836621	I	Ogliarola
ogliarola_Ri_190	9-oxoODE	1200,101658	R	Ogliarola
ogliarola_Ri_191	9-oxoODE	4404,206568	R	Ogliarola
ogliarola_Ri_192	9-oxoODE	3240,918957	R	Ogliarola

ogliarola_Ri_193	9-oxoODE	2815,768058	R	Ogliarola
ogliarola_Ri_194	9-oxoODE	4053,805937	R	Ogliarola
ogliarola_Ri_195	9-oxoODE	7916,046414	R	Ogliarola
ogliarola_sana_154	9-oxoODE	6257,501775	H	Ogliarola
ogliarola_sana_155	9-oxoODE	3217,482845	H	Ogliarola
ogliarola_sana_156	9-oxoODE	4490,356533	H	Ogliarola
ogliarola_sana_157	9-oxoODE	4845,199953	H	Ogliarola
ogliarola_sana_158	9-oxoODE	11130,35605	H	Ogliarola
ogliarola_sana_159	9-oxoODE	8232,86366	H	Ogliarola
cellina_infetti_200	9-oxoOTrE	2213,489108	I	Cellina
cellina_infetti_201	9-oxoOTrE	1731,635393	I	Cellina
cellina_infetti_208	9-oxoOTrE	753,407799	I	Cellina
cellina_infetti_209	9-oxoOTrE	911,9957454	I	Cellina
cellina_infetti_214	9-oxoOTrE	1008,090995	I	Cellina
cellina_infetti_217	9-oxoOTrE	1290,532374	I	Cellina
cellina_Ri_184	9-oxoOTrE	1541,372369	R	Cellina
cellina_Ri_185	9-oxoOTrE	3329,911549	R	Cellina
cellina_Ri_186	9-oxoOTrE	2444,521535	R	Cellina
cellina_Ri_187	9-oxoOTrE	3509,698796	R	Cellina
cellina_Ri_196	9-oxoOTrE	3860,715444	R	Cellina
cellina_Ri_197	9-oxoOTrE	1183,663189	R	Cellina
cellina_sani_169	9-oxoOTrE	4869,549923	H	Cellina
cellina_sani_170	9-oxoOTrE	5003,140633	H	Cellina
cellina_sani_171	9-oxoOTrE	12303,35252	H	Cellina
cellina_sani_172	9-oxoOTrE	16177,9961	H	Cellina
cellina_sani_176	9-oxoOTrE	2355,577294	H	Cellina
cellina_sani_179	9-oxoOTrE	2832,700858	H	Cellina
ogliarola_infetti_202	9-oxoOTrE	4426,643885	I	Ogliarola
ogliarola_infetti_203	9-oxoOTrE	3186,572021	I	Ogliarola
ogliarola_infetti_206	9-oxoOTrE	2382,750771	I	Ogliarola
ogliarola_infetti_207	9-oxoOTrE	10304,25595	I	Ogliarola
ogliarola_infetti_213	9-oxoOTrE	21212,18964	I	Ogliarola
ogliarola_infetti_216	9-oxoOTrE	1041,175293	I	Ogliarola
ogliarola_Ri_190	9-oxoOTrE	1172,614676	R	Ogliarola
ogliarola_Ri_191	9-oxoOTrE	3242,408851	R	Ogliarola
ogliarola_Ri_192	9-oxoOTrE	2455,520018	R	Ogliarola
ogliarola_Ri_193	9-oxoOTrE	2585,595539	R	Ogliarola
ogliarola_Ri_194	9-oxoOTrE	3551,615008	R	Ogliarola
ogliarola_Ri_195	9-oxoOTrE	7544,602346	R	Ogliarola
ogliarola_sana_154	9-oxoOTrE	2813,764353	H	Ogliarola
ogliarola_sana_155	9-oxoOTrE	2258,136946	H	Ogliarola
ogliarola_sana_156	9-oxoOTrE	3203,081324	H	Ogliarola
ogliarola_sana_157	9-oxoOTrE	3463,090595	H	Ogliarola
ogliarola_sana_158	9-oxoOTrE	8358,924643	H	Ogliarola
ogliarola_sana_159	9-oxoOTrE	4869,670259	H	Ogliarola
cellina_infetti_200	SA	1254,535338	I	Cellina

cellina_infetti_201	SA	1302,863723	I	Cellina
cellina_infetti_208	SA	5441,01393	I	Cellina
cellina_infetti_209	SA	4313,354909	I	Cellina
cellina_infetti_214	SA	1557,175396	I	Cellina
cellina_infetti_217	SA	1697,719477	I	Cellina
cellina_Ri_184	SA	10829,9547	R	Cellina
cellina_Ri_185	SA	7064,045405	R	Cellina
cellina_Ri_186	SA	24504,37885	R	Cellina
cellina_Ri_187	SA	17467,19992	R	Cellina
cellina_Ri_196	SA	15387,67059	R	Cellina
cellina_Ri_197	SA	10544,21942	R	Cellina
cellina_sani_169	SA	2209,548131	H	Cellina
cellina_sani_170	SA	1552,455018	H	Cellina
cellina_sani_171	SA	1714,945761	H	Cellina
cellina_sani_172	SA	906,9515195	H	Cellina
cellina_sani_176	SA	982,6593694	H	Cellina
cellina_sani_179	SA	507,4704809	H	Cellina
ogliarola_infetti_202	SA	13555,90511	I	Ogliarola
ogliarola_infetti_203	SA	6423,546905	I	Ogliarola
ogliarola_infetti_206	SA	6589,537833	I	Ogliarola
ogliarola_infetti_207	SA	22042,39119	I	Ogliarola
ogliarola_infetti_213	SA	33924,41589	I	Ogliarola
ogliarola_infetti_216	SA	3860,642227	I	Ogliarola
ogliarola_Ri_190	SA	25196,21088	R	Ogliarola
ogliarola_Ri_191	SA	11534,8053	R	Ogliarola
ogliarola_Ri_192	SA	17969,12874	R	Ogliarola
ogliarola_Ri_193	SA	7616,999559	R	Ogliarola
ogliarola_Ri_194	SA	32243,54331	R	Ogliarola
ogliarola_Ri_195	SA	42879,9753	R	Ogliarola
ogliarola_sana_154	SA	5084,844998	H	Ogliarola
ogliarola_sana_155	SA	4802,606682	H	Ogliarola
ogliarola_sana_156	SA	10210,28374	H	Ogliarola
ogliarola_sana_157	SA	12496,49567	H	Ogliarola
ogliarola_sana_158	SA	10041,08056	H	Ogliarola
ogliarola_sana_159	SA	4993,608832	H	Ogliarola

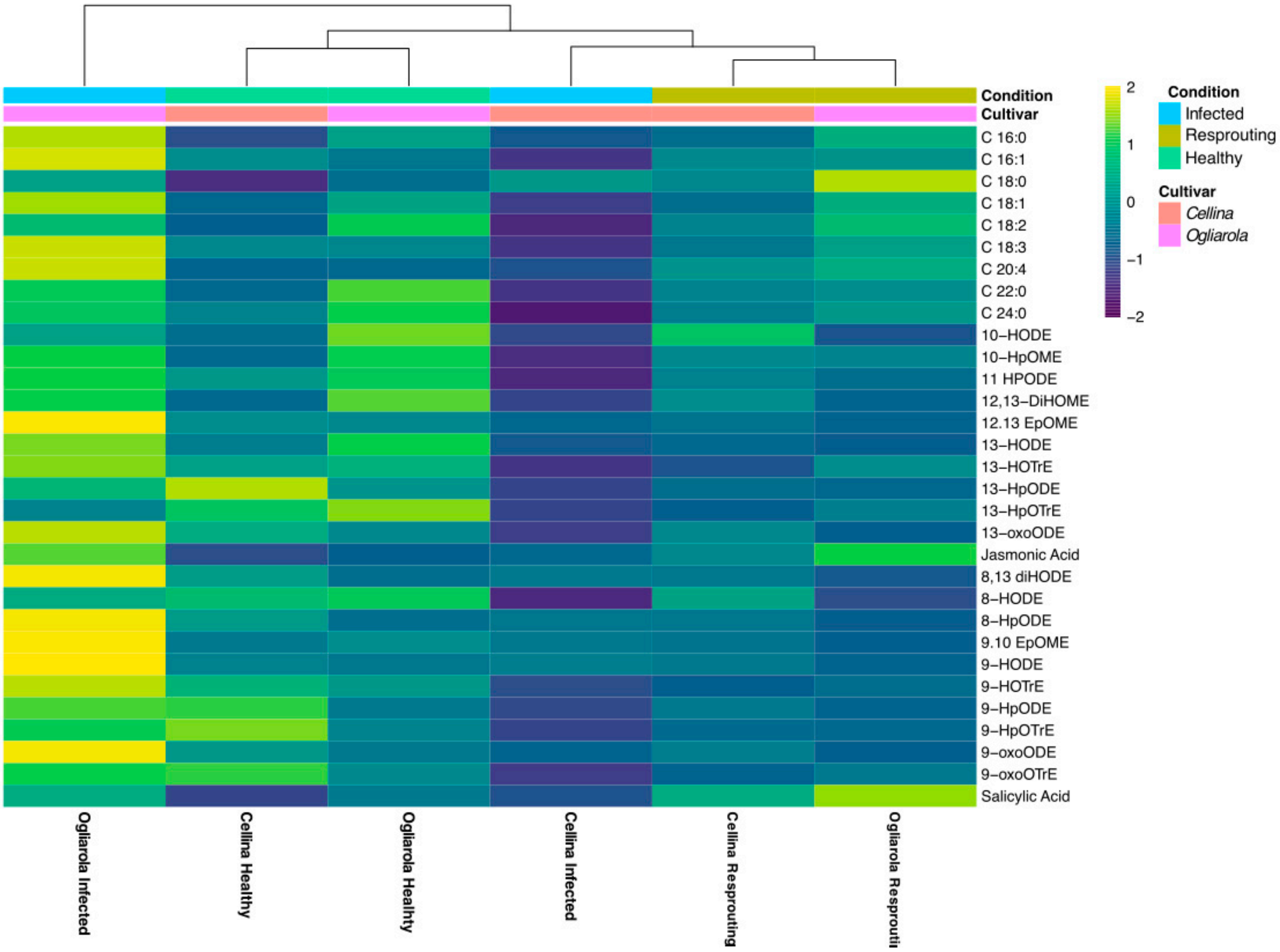

Supplementary Figure S1. Heatmap of all lipid entities analyzed by LC-MS/MS. Figure reports free fatty acids and oxylipins extracted and analysed from the cultivars Cellina and Ogliarola in Healthy, Infected and Resprouting samples, that were clustered by state of healthy state, cultivar and lipid entities. Palmitic acid (C16:0), palmitoleic acid (C16:1), stearic acid (C18:0), oleic acid (C18:1), linoleic acid (C18:2), linolenic acid (C18:3), arachidonic acid (C20:4), behenic acid (22:0), lignoceric acid (C24:0), hydroxyoctadecenoic acid (HODE), hydroperoxyoctamonoenoic acid (HpOME), hydroperoxyoctadienoic acid (HpODE), dihydroxyoctamonoenoic acid (DiHOME), epoxyoctamonoenoic acid (epOME), hydroxyoctatrienoic acid (HOTrE), hydroperoxyoctatrienoic acid (HpOTrE), oxo-octadecenoic acid, (OxoODE), and oxo-octadecatrienoic acid (OxoOTrE). Notation of the FAs and oxylipins (OM/D/TrE) is reported as the carbon number (CN) and the number of double bond (DB) equivalents.

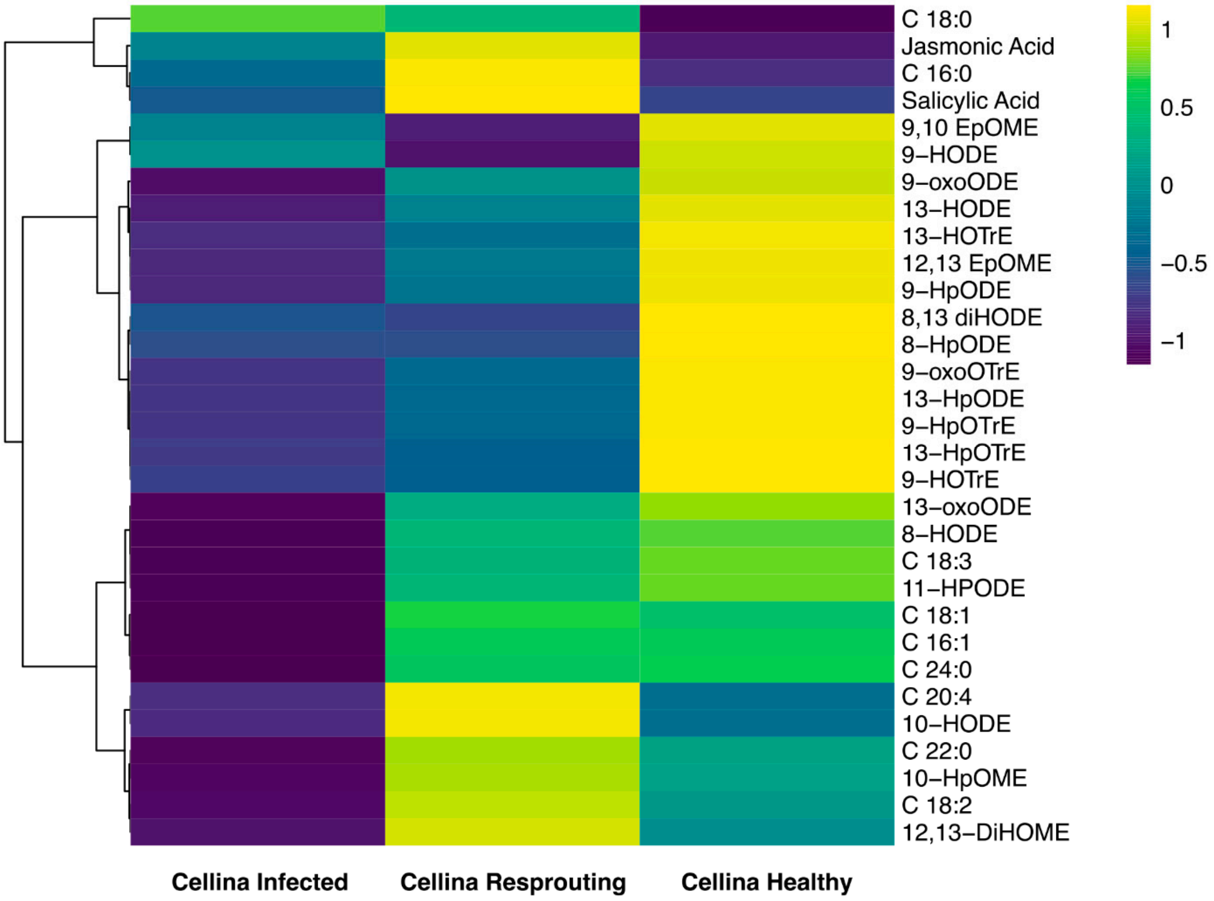

Supplementary Figure S2. Heatmap of all free fatty acids and oxylipins analyzed by LC-MS/MS in the Healthy, Infected, and Resprouting samples of the cultivar Cellina. The heatmaps reported the samples divided per healthy state. Palmitic acid (C16:0), palmitoleic acid (C16:1), stearic acid (C18:0), oleic acid (C18:1), linoleic acid (C18:2), linolenic acid (C18:3), arachidonic acid (C20:4), behenic acid (22:0), lignoceric acid (C24:0), hydroxyoctadecenoic acid (HODE), hydroperoxyoctamonoenoic acid (HpOME), hydroperoxyoctadienoic acid (HpODE), dihydroxyoctamonoenoic acid (DiHOME), epoxyoctamonoenoic acid (epOME), hydroxyoctatrienoic acid (HOTrE), hydroperoxyoctatrienoic acid (HpOTrE), oxo-octadecenoic acid, (OxoODE), and oxo-octadecatrienoic acid (OxoOTrE). Notation of the FAs and oxylipins (OM/D/TrE) is reported as the carbon number (CN) and the number of double bond (DB) equivalents.

layout.

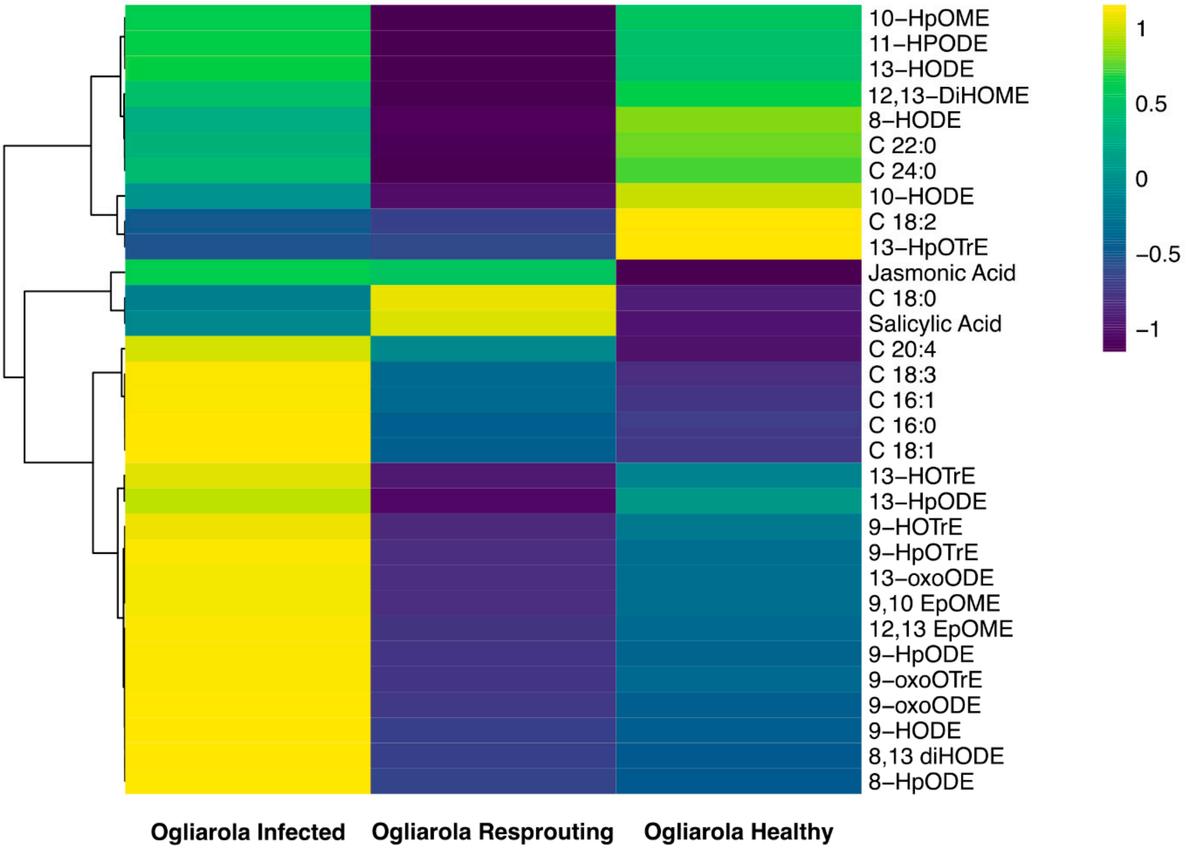

Supplementary Figure S3. Heatmap of all free fatty acid and oxylipins analyzed by LC-MS/MS in healthy, infected, and resprouting samples of the cultivar Ogliarola. Palmitic acid (C16:0), palmitoleic acid (C16:1), stearic acid (C18:0), oleic acid (C18:1), linoleic acid (C18:2), linolenic acid (C18:3), arachidonic acid (C20:4), behenic acid (22:0), lignoceric acid (C24:0), hydroxyoctadecenoic acid (HODE), hydroperoxyoctamonoenoic acid (HpOME), hydroperoxyoctadienoic acid (HpODE), dihydroxyoctamonoenoic acid (DiHOME), epoxyoctamonoenoic acid (epOME), hydroxyoctatrienoic acid (HOTrE), hydroperoxyoctatrienoic acid (HpOTrE), oxo-octadecenoic acid, (OxoODE), and oxo-octadecatrienoic acid (OxoOTrE). Notation of the FAs and oxylipins (OM/D/TrE) is reported as the carbon number (CN) and the number of double bond (DB) equivalents. In figure the heatmaps reported the samples divided per healthy state.

Supplementary Figure S4. The total amount of oxylipins [(hydroxyoctadecenoic acid (HODE), hydroperoxyoctamonoenoic acid (HpOME), hydroperoxyoc-tadienoic acid (HpODE), dihydroxyoctamonoenoic acid (DiHOME), epoxyoctamonoenoic acid (epOME), hydroxyoctatrienoic acid (HOTrE), hydroperoxyoctatrienoic acid (HpOTrE), oxo-octadecenoic acid, (OxoODE), and oxo-octadecatrienoic acid (OxoOTrE)] expressed as relative abundance normalized with ISTD (9-HODE) in R, I and H. H: indicates negative to XFP olive trees; I indicates positive to XFP and OQDS symptomatic olive trees; R indicates positive to XFP and resprouting olive tree. T test was used to compare samples mean values.

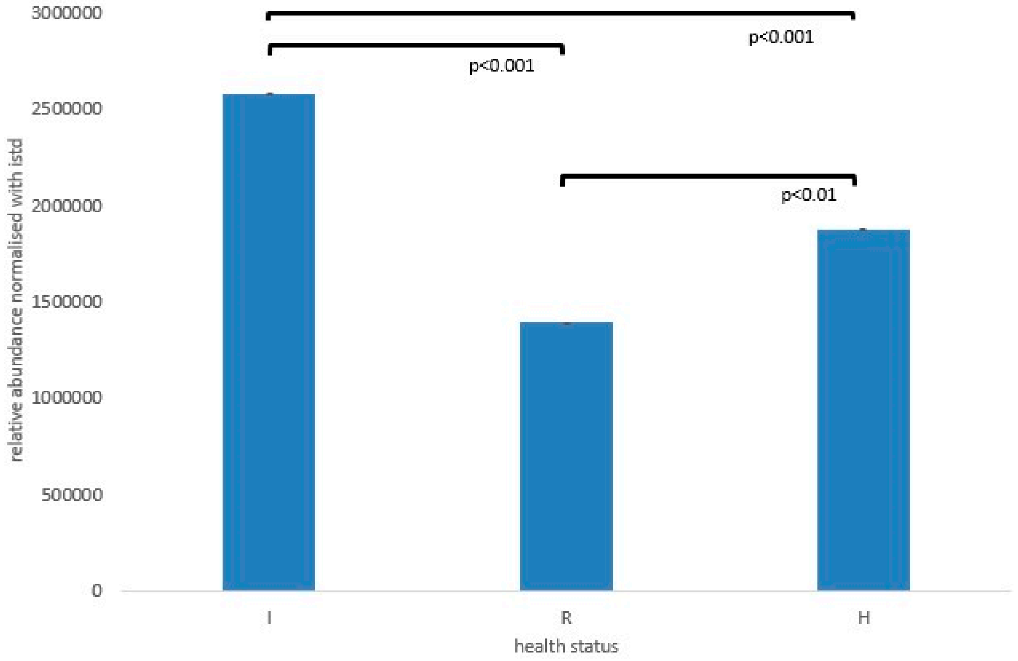

Supplementary Figure S5. The olive groves where the XFP-positive resprouts were collected (A). One of the olive trees of the Cellina di Nardò variety (B) and one of the Ogliarola Salentina variety (C) that were selected for sampling.

A)

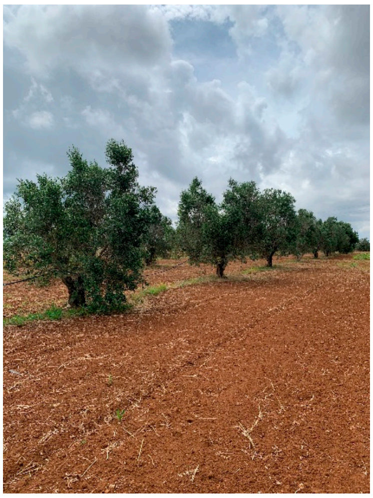

B)

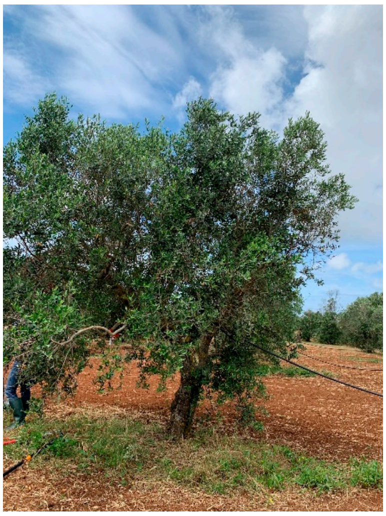

C)

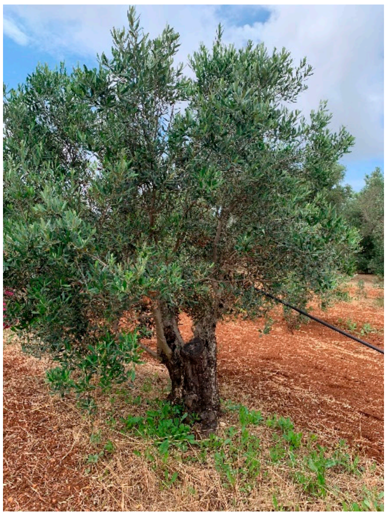

Supplement: Supplementary file 1 [file plants-13-02186-s001.zip › Supplementary_table_figure.pdf]
